# Nanofluidic Trapping of Faceted Colloidal Nanocrystals for Parallel Single-Particle Catalysis

**DOI:** 10.1021/acsnano.2c06505

**Published:** 2022-09-02

**Authors:** Sune Levin, Sarah Lerch, Astrid Boje, Joachim Fritzsche, Sriram KK, Henrik Ström, Kasper Moth-Poulsen, Henrik Sundén, Anders Hellman, Fredrik Westerlund, Christoph Langhammer

**Affiliations:** † Department of Biology and Biological Engineering, Chalmers University of Technology; SE-412 96 Gothenburg, Sweden; ‡ Department of Chemistry and Chemical Engineering, Chalmers University of Technology; SE-412 96 Gothenburg, Sweden; § Department of Physics, Chalmers University of Technology; SE-412 96 Gothenburg, Sweden; ∥ Department of Mechanics and Maritime Sciences, Chalmers University of Technology; SE-412 96 Gothenburg, Sweden; ⊥ Department of Energy and Process Engineering, Norwegian University of Science and Technology; NO-7034 Trondheim, Norway; # Institute of Materials Science of Barcelona, ICMAB-CSIC, Bellaterra, ES-08193 Barcelona, Spain; ¶ Catalan Institution for Research and Advanced Studies, ICREA; ES-08010 Barcelona, Spain; ○ Department of Chemistry & Molecular Biology, University of Gothenburg; SE-412 96 Gothenburg, Sweden; △ Competence Centre for Catalysis, Chalmers University of Technology; SE-412 96 Gothenburg, Sweden

**Keywords:** nanoparticle trapping, single nanoparticle catalysis, nanofluidics, fluorescence microscopy, colloidal Au nanocrystals, first-principles calculations

## Abstract

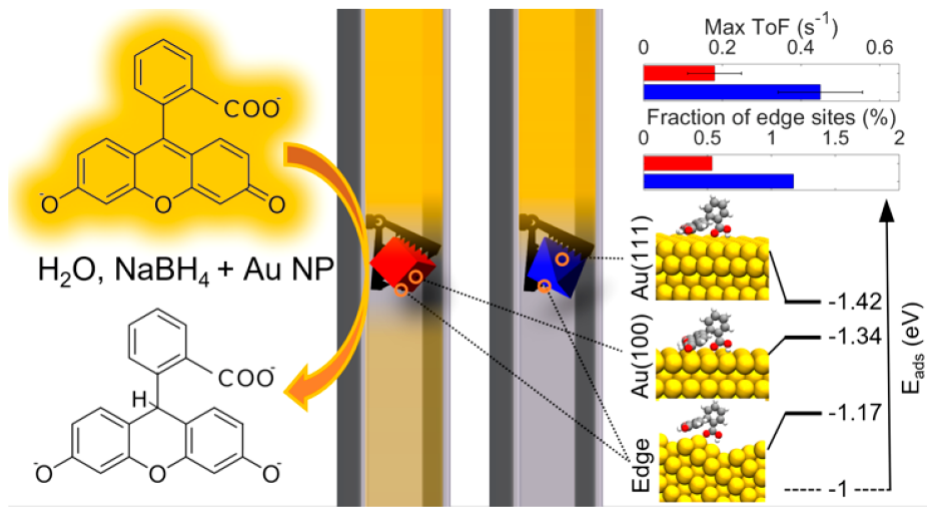

Catalyst activity can depend distinctly on nanoparticle size and shape. Therefore, understanding the structure sensitivity of catalytic reactions is of fundamental and technical importance. Experiments with single-particle resolution, where ensemble-averaging is eliminated, are required to study it. Here, we implement the selective trapping of individual spherical, cubic, and octahedral colloidal Au nanocrystals in 100 parallel nanofluidic channels to determine their activity for fluorescein reduction by sodium borohydride using fluorescence microscopy. As the main result, we identify distinct structure sensitivity of the rate-limiting borohydride oxidation step originating from different edge site abundance on the three particle types, as confirmed by first-principles calculations. This advertises nanofluidic reactors for the study of structure–function correlations in catalysis and identifies nanoparticle shape as a key factor in borohydride-mediated catalytic reactions.

In catalysis, microreactors have become a workhorse for tailoring catalytic reactions, for catalyst material discovery, and for studying catalytic processes.^1−3^ Such microreactors have 10–100s of micrometer lateral dimensions. Parallel to this development, nanofluidics has evolved and downsized the control of fluids to the nanoscale.^4,5^ These efforts were predominantly driven by bionanotechnology, with the ultimate goal of studying individual biomolecules.^6,7^ Projected onto catalysis, nanofluidics offers interesting prospects, such as controlling mass transport to or from individual nanosized objects,^8^ mimicking nanoconfinement imposed by nano- and mesopores of catalyst support materials,^9^ and the accumulation of molecular products on a single catalyst particle to concentrations high enough for detection.^10^ Therefore, nanofluidic structures are of high relevance for application in single-particle catalysis that aims to map structure–function correlations beyond the ensemble average.^11−16^

Here, we demonstrate the trapping of individual shape-selected colloidal Au nanocrystals from solution at well-defined positions inside an array of 100 parallel nanofluidic channels. This platform ensures identical reaction conditions for all particles, since each is isolated in its own channel, and enables parallel quantitative activity monitoring from tens of individual particles using fluorescence microscopy. Furthermore, the platform facilitates the direct comparison of single particles with different predetermined sizes and shapes in a single experiment, thereby eliminating cross-experiment errors inherent to standard sequential measurements. In contrast to our previous design, where we studied polycrystalline nanoparticles fabricated by electron beam lithography,^10^ we are here able to trap and study single crystalline colloidal nanoparticles synthesized in solution and with highly defined shape and surface facets. We apply this platform to study the reduction of fluorescein by sodium borohydride over single Au nanocrystals, which is a relevant model system for borohydride-mediated catalytic reactions in organic synthesis,^17,18^ for hydrogen production,^19^ and for the oxidation of borohydride in direct borohydride fuel cells (DBFCs).^20,21^ By consecutively trapping colloidal Au faceted spheres, cubes, and octahedra in the 70–100 nm size range in a single device and measuring their activity, we reach industrially relevant^22^ turnover frequencies (ToFs) between 0.2 and 0.6 s^–1^ per surface atom. Furthermore, we reveal a distinct structure sensitivity of the reaction with octahedra being most active. We attribute this to their large fraction of edge sites that aids catalyst activation from a fluorescein-poisoned state and increases the rate of borohydride oxidation, as revealed by first-principles calculations.

## Results and Discussion

**Figure 1:**
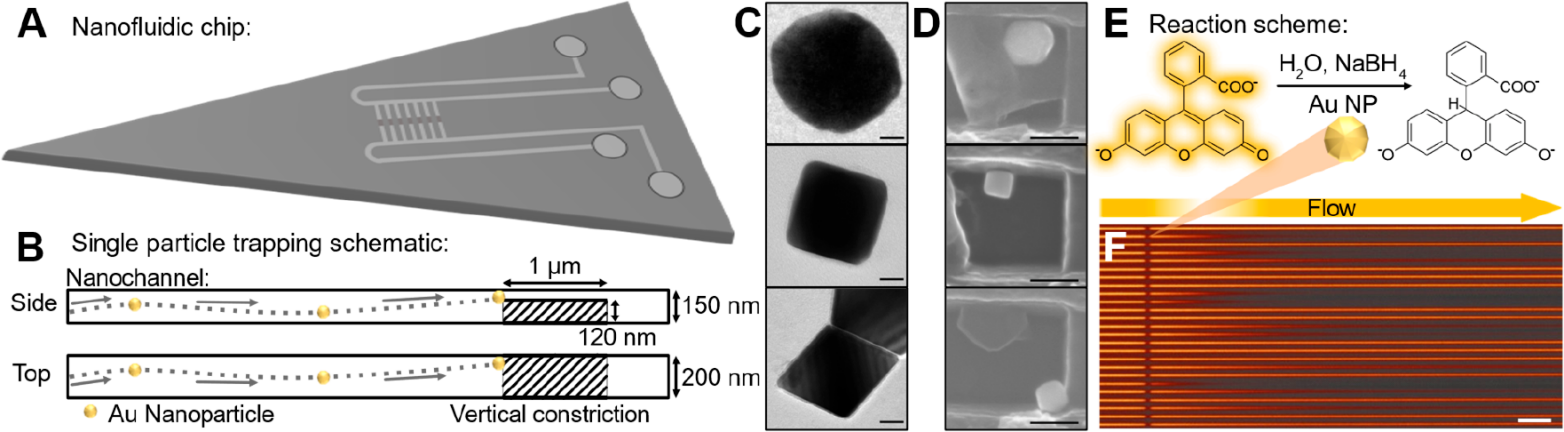
Experimental setup, colloidal nanocrystals, and reaction and readout scheme. (A) Schematic of the nanofluidic chip with four in-/outlets connected to microchannels that contact an array of 100 parallel nanochannels functionalized with particle traps in their central region. (B) Schematic depiction of the particle trap composed of a 120 × 1000 nm^2^ vertical constriction nanofabricated in the center of the nanochannel. This design enables the trapping of objects larger than the 30 nm gap between the constriction and the channel wall. (C) Representative TEM images of the three types of Au colloidal crystals investigated, taken directly after synthesis—from top: faceted sphere, cube, octahedron. Scale bars = 20 nm. (D) SEM images of trapped nanocrystals—from top: faceted sphere, cube, octahedron in a trap. Scale bars = 100 nm. (E) Schematic for the reduction of fluorescein with sodium borohydride on an Au nanoparticle. The reduced fluorescein is nonemissive. (F) Fluorescence microscopy image of 25 nanochannels during an experiment. Scale bar = 10 μm.

Our nanofluidic devices are comprised of in- and outlet microchannels that contact the array of 100 parallel nanochannels, each of which is 150 nm high, 200 nm wide and 350 μm long (Figure 1A). They were micro- and nanofabricated into an oxidized silicon wafer sealed with a fusion-bonded glass lid, as outlined in Materials and Methods. As the key feature, we nanofabricated a vertical constriction that is 120 nm high, 200 nm wide, and 1 μm long and acts as a physical trap for nanoparticles larger than 30 nm in the center of each nanochannel (Figure 1B). We utilized this feature to trap three different kinds of citrate- and/or PVP-stabilized colloidal Au nanoparticles, i.e., faceted spheres with nominal 100 nm diameter, cubes with 76 nm, and octahedra with 74 nm average side length (Figure 1C). The trapping of these nanocrystals was enabled by flushing them through the fluidic system in aqueous suspension at a concentration of ∼10^9^ particles per mL. In this way, we could successfully trap individuals of all three particle types, as corroborated by scanning electron microscopy (SEM) images of traps obtained after removal of a lid purposely bonded with polysilsesquioxane (PSQ)^23,24^ to enable reopening (Figure 1D; Figures S1, S2, and Supplementary Text Section 1).

We investigated the catalytic activity of the trapped particles in the reduction of fluorescein (Figure 1E) by reversing the flow direction. By then measuring the catalytic reaction-induced fluorescence intensity reduction downstream of the particles (Figure 1F, Supplementary Text Section 2), we extracted the ToF per surface atom of each particle in a trap^10^ and guaranteed that it was unaffected by nonspecifically attached particles present on the (former) inlet side of the chip.

For the colloidal nanocrystals to be trapped in the designated place, it is critical that they do not attach to the walls of the fluidic system before reaching the traps. This can be achieved by repulsive electrostatic interactions between the particles and channels. However, since the most common ligands used for shape-controlled synthesis, such as hexadecyltrimethylammonium chloride (CTAC) used here (Materials and Methods), result in positively charged particles^25−27^ and since the charge of a silica surface is ∼ −60 mV in water at neutral pH,^28^ we performed a ligand exchange. This yielded mixed citrate- and PVP-stabilized Au cubes and octahedra with a surface charge of approximately −30 mV.^26^ The Au spheres were used as purchased with citrate stabilization that has a similar negative charge.

**Figure 2:**
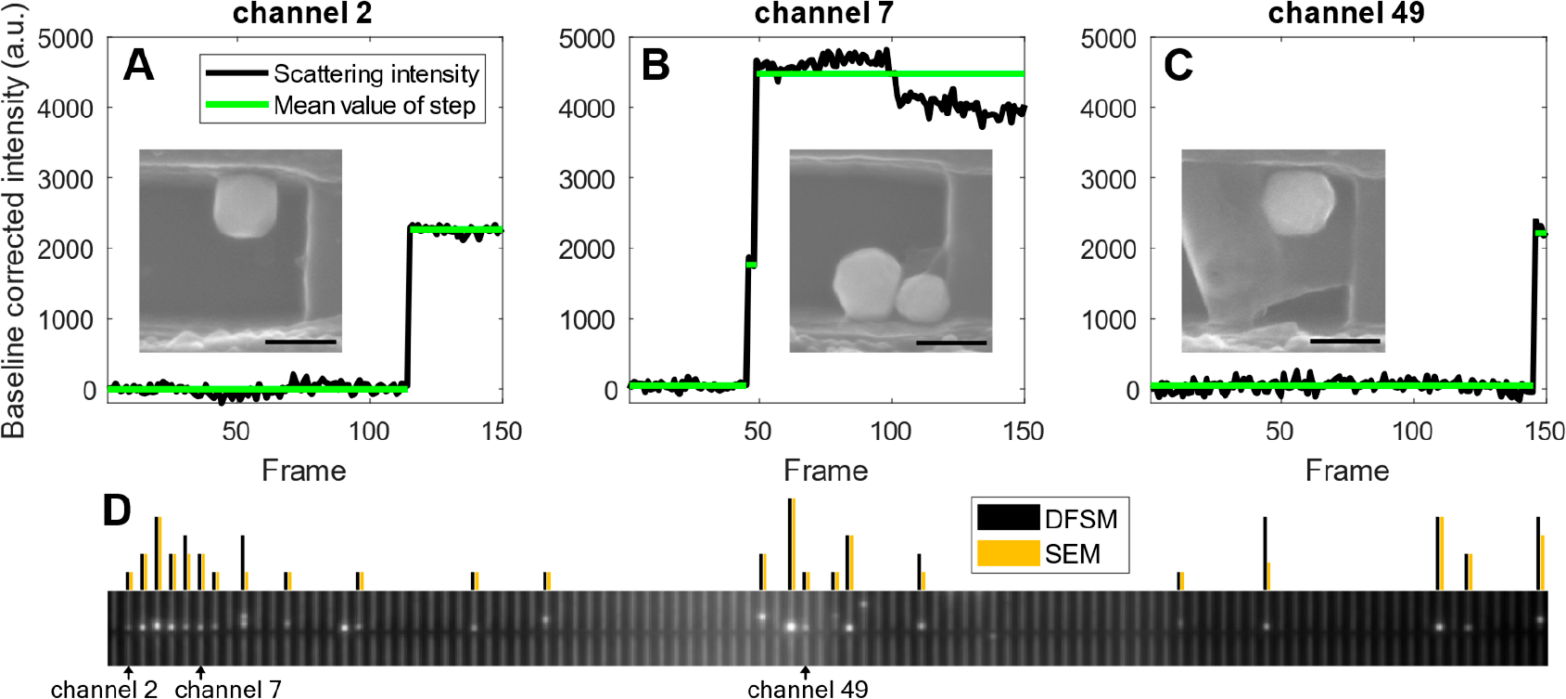
Dark-field scattering microscopy (DFSM) of single-particle trapping. Scattering intensity profiles (black) for three different nanochannels upon trapping of a single (A), two (B), and a single (C) Au faceted sphere(s), respectively. The green lines depict a step-function fitted to the raw data for the automatic detection of a trapped particle. Insets in (A–C) show SEM images of the nanoparticle trap where the intensity profile was recorded, as taken after removal of the PSQ-bonded lid, which corroborates the direct correspondence between measured step size and the number of trapped particles. (D) DFSM image of all the 100 nanochannels at the position of the traps, in which the bright spots indicate the presence of one or more trapped Au faceted sphere(s). The bars depicted above each channel in the scattering image indicate the number of trapped particles, as determined by SEM and DFSM.

**Figure 3:**
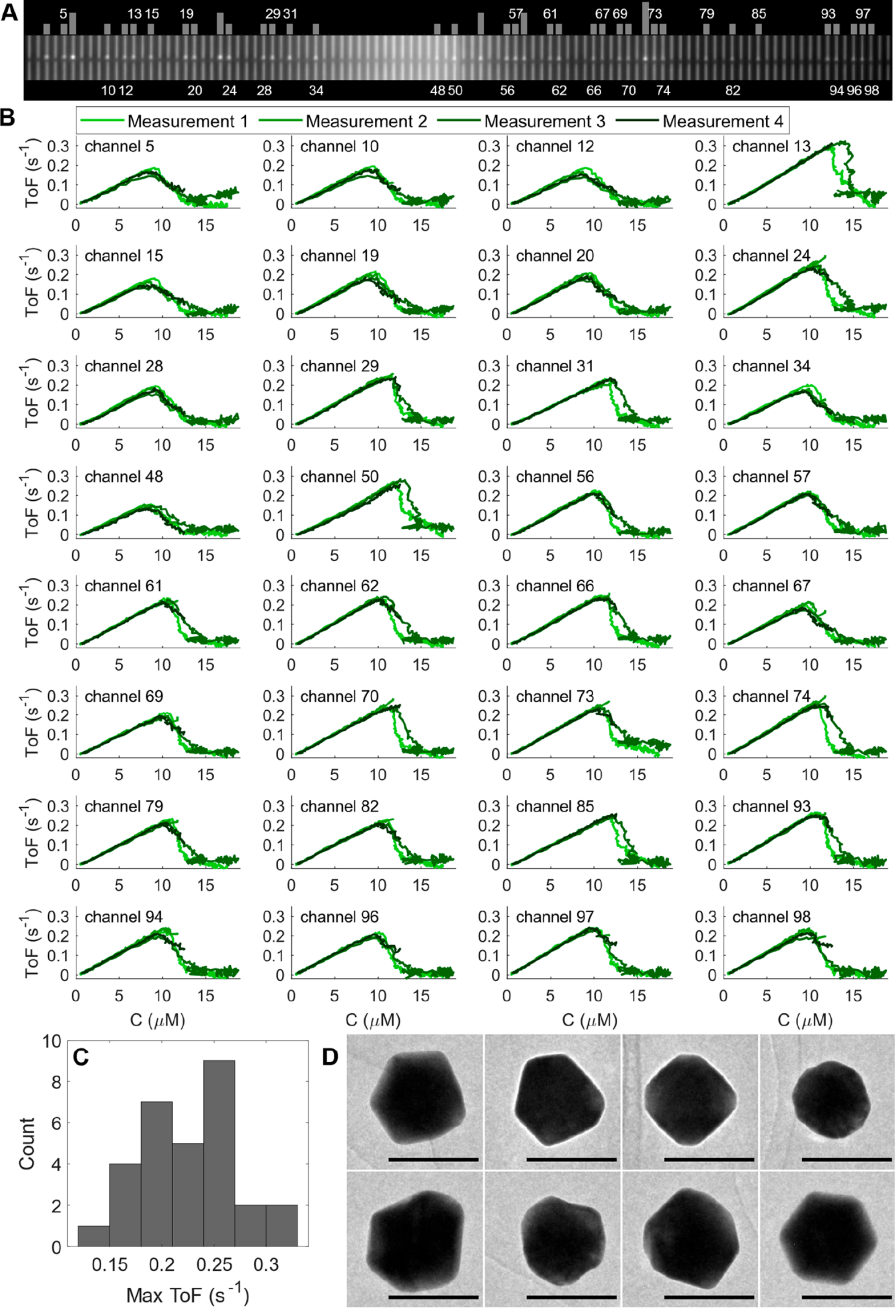
Fluorescein reduction with sodium borohydride on individual 100 nm colloidal Au faceted spheres. (A) DFSM image of the 100 nanochannels on the chip, where bright spots indicate trapped particles. The gray bars indicate the number of particles, and for channels containing a single Au particle, the channel number is displayed. (B) ToFs per surface atom determined from 4 subsequent measurements for 32 individual 100 nm Au faceted spheres upon subsequent fluorescein concentration sweeps starting at *C*_start_ = 18 μM. (C) Histogram of the maximal ToF distribution for the particles in (B). (D) TEM images of a representative selection of Au faceted spheres taken on an open surface revealing their widely varying structure. Scale bars = 100 nm.

We identified the traps with single particles versus those with multiple particles by recording the trapping procedure using single-particle dark-field scattering microscopy (DFSM)^16^ (Video S1). Specifically, we extracted an intensity time trace for each trap during the influx of nanoparticles, which resulted in intensity step functions that signal the arrival of a particle because of their large scattering cross sections provided by the excitation of their intrinsic localized surface plasmon resonance (Figure 2A–C). We then analyzed these intensity traces to count the number of particles that arrived in each nanochannel. This approach was corroborated when we again employed a PSQ-bonded chip, which enabled SEM imaging of the traps after particle capture (Figure 2A–C insets and Figure S3), to directly compare the number of particles counted for each trap using the DFSM approach and the direct SEM imaging with excellent agreement (Figure 2D and Figure S4). Furthermore, this analysis revealed that trapping particles up to an occupation of ∼50% of the nanochannels generally results in ∼30 out of 100 nanochannels containing a single particle and a few channels containing several particles, which is in good agreement with a Poisson distribution for particles randomly entering the channels with equal probability (Figure 3A, Video S1). We also note that neither flow nor pressure drop across the channel is significantly affected by particles attaching at the trap because of the overall length of the channels.^10^

As a further aspect, we highlight that it is important that the particles, once trapped, stay in place after flow reversal for the subsequent catalysis experiments. Here, we could rely on their localization within the recirculation region behind the vertical constriction during reversed flow. Second, we minimized the electrostatic repulsion between the channel walls and the particles by flushing a 5 M NaCl solution to reduce the Debye–Hückel screening length^29,30^ after the trapping, followed by ligand removal from the trapped particles using a 20 min flush of 4.3 wt % ammonia and 4.3 wt % hydrogen peroxide in milli-Q water.^10^ In this way, we ensured that we assessed the intrinsic activity of the metallic surfaces, rather than the effect of different ligands.^31,32^

As the first catalysis experiment on trapped Au nanocrystals, we simultaneously examined the reduction of fluorescein by sodium borohydride on 32 individual nanospheres with a nominal diameter of 100 nm (Figure 3A). Specifically, we reduced the fluorescein concentration in the incoming reactant solution from 18 μM to 0 μM while keeping the borohydride concentration constant at 50 mM. The extraction of the correspondingly measured ToF for each particle (Supplementary Text Section 2) and the plotting of it versus incoming fluorescein concentration, *C*, revealed a distinct transition from a surface-poisoned regime at high *C*, via a maximum in activity, to a mass-transport limited regime characterized by a linear dependence of ToF on *C* at low fluorescein concentrations (Figure 3B). Furthermore, we noticed that both the point and shape of the transition between the different reaction regimes varied between particles, despite qualitatively similar behavior (Figure 3C). Notably, this single-particle-specific behavior was highly reproducible in subsequent experiments (different colors in Figure 3B) and likely reflects the distribution of surface sites on these multifaceted Au nanoparticles (Figure 3D and Figure S5), thus suggesting a structure sensitivity of the reaction.

**Figure 4:**
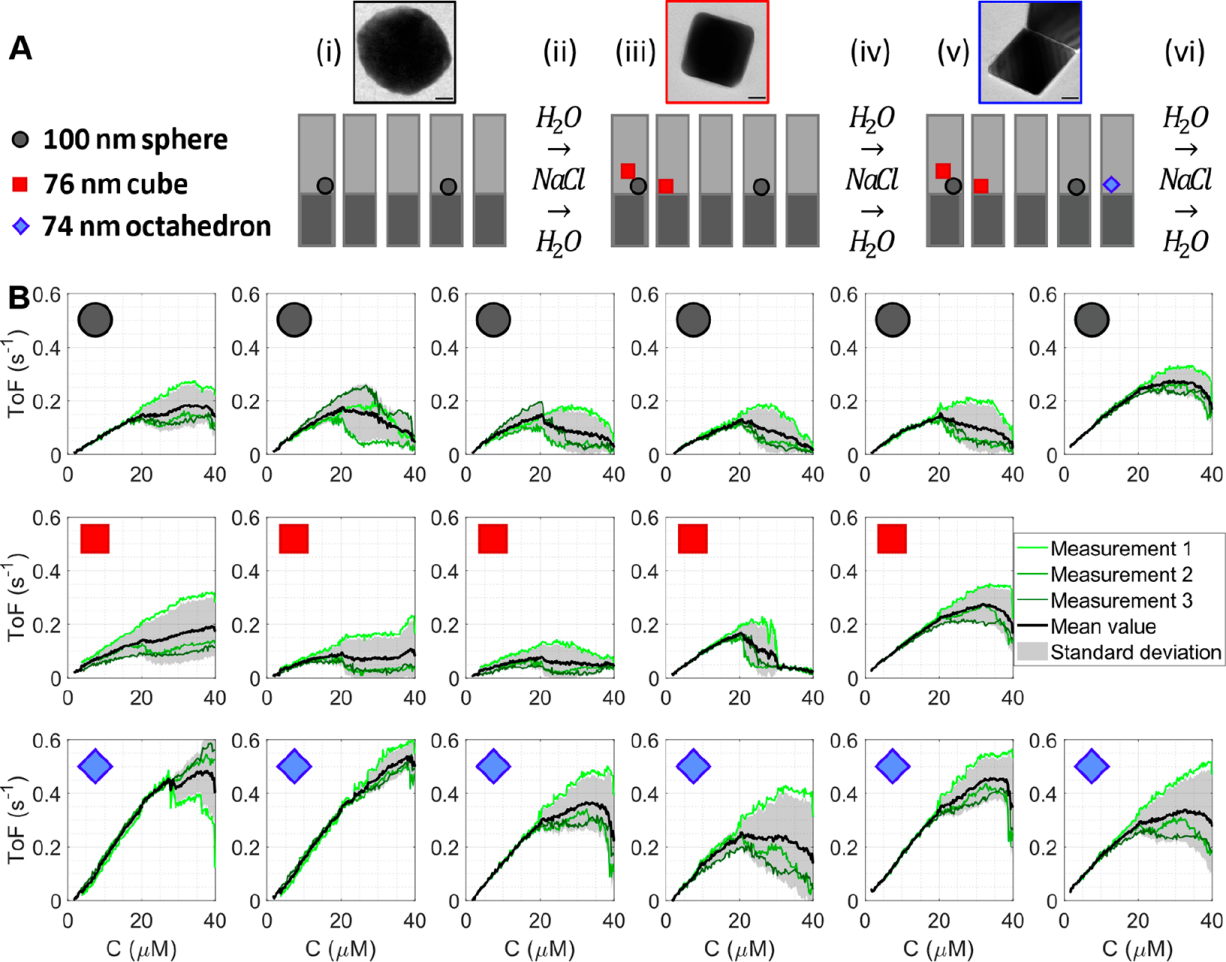
Fluorescein reduction with sodium borohydride on individual Au faceted spheres, cubes, and octahedra. (A) Schematic of the process flow during the consecutive trapping of colloidal particles with different shapes: trapping of (i) 100 nm Au faceted spheres, (iii) 76 nm Au cubes, (v) 74 nm Au octahedra, and (ii, iv, vi) flushing with NaCl solution and water. (B) ToF per surface atom for trapped individual Au faceted spheres, cubes, and octahedra, measured simultaneously in the same chip upon three subsequent fluorescein concentration sweeps starting at *C*_start_ = 40 μM.

We exploited the ability of the fluidic chips to host different colloidal nanocrystal types with distinct predetermined shapes and faceting to quantitatively investigate potential structure sensitivity, hinted at by the experiments on the faceted spheres, and to evaluate their activity in one and the same experiment. For this purpose, we trapped 100 nm Au faceted spheres, 76 nm Au cubes, and 74 nm Au octahedra in a single chip (Figures S5–S8 and Table S1). This becomes possible by iterating the trapping procedure in a single device several times with different types of particles (Figure 4A). We again used DFSM to detect and count the particles, which enabled establishing a distribution of each particle type inside the 100 channels separately (Figure S9). This analysis revealed the successful trapping of six individual spheres, five cubes, and six octahedra to be evaluated for their catalytic activity.

**Figure 5:**
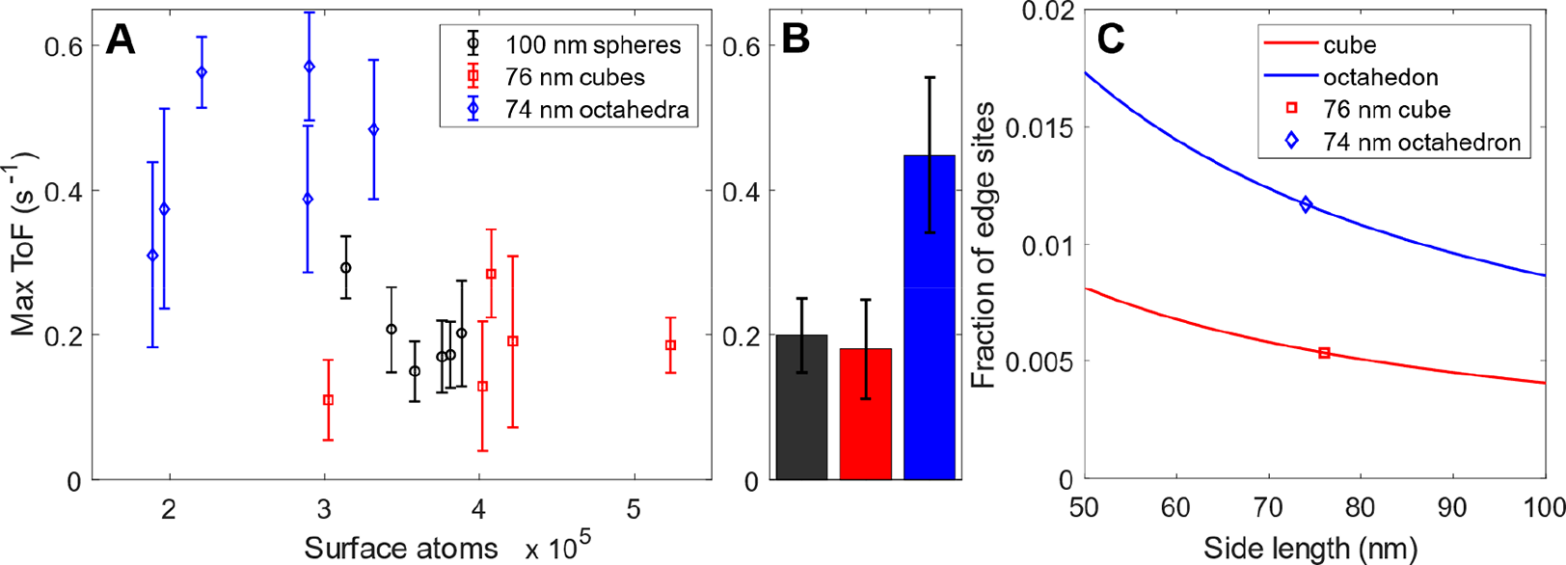
Structure sensitivity of fluorescein reduction. (A) Maximal ToF of the reaction traces depicted in Figure 4B for Au faceted spheres, cubes, and octahedra, plotted as a function of number of estimated surface atoms of each particle (Supplementary Text Section 4). Error bars are the standard deviation between three measurements. (B) Average values and standard deviation (error bars) between the six Au faceted spheres, the five cubes, and the six octahedra plotted in (A). (C) Fraction of edge sites as a function of particle side length for cubes and octahedra for side lengths between 50 and 100 nm. The octahedron displays nearly twice the fraction of edge sites for all side lengths compared with the cube.

The activity of the three different populations of Au nanocrystals in the device was simultaneously assessed by scanning the fluorescein concentration in three subsequent measurements to examine the transition between the surface-poisoned and the mass-transport limited regime. We started at 40 μM fluorescein with a constant sodium borohydride concentration of 50 mM. Extraction of the correspondingly measured ToF for each particle revealed generally similar reaction profiles as in the experiment with only 100 nm faceted spheres (Figure 4B), albeit with somewhat different onset concentration (compare with Figure 3B). This highlights the importance of assessing the different particles simultaneously and in the same fluidic system to ensure identical reaction conditions since these kind of systems generally express day-to-day variations (Supplementary Text Section 3 and Figure S10). As a general trend and a first key result, we noticed that the maximum ToF is higher for the octahedra than for the cubes and the faceted spheres. Furthermore, the particles also display distinct variations in activity within each particle type. Importantly, while the activity for each particle varied somewhat between different measurements, and the maximum ToF decreased for all particles from a mean value of 0.29 to 0.1 s^–1^, the relative trends in particle-specific activity remained the same across multiple subsequent measurement series (Figure S11–S16 and compare Figure S12 with Figure 5). This corroborates the significance of these interparticle differences and implies that they likely reflect the particle-specific structure.

The activity profiles obtained for the three different particle types across the transition from the surface-poisoned to the mass-transport limited regime were further analyzed by plotting the maximum ToF for each particle as a function of the total number of surface atoms in each particle (Figure 5A), which we derived from the DFSM intensity measured during particle trapping (Supplementary Text Section 4 and Figure S17). In this representation, we can distribute the particles with respect to their estimated size to reveal potential size dependencies within the population of a specific particle type. As the main result, we found a generally significantly higher maximal ToF for the octahedra compared with the cubes and the spheres (Figure 5B) but no significant size dependence within each population, which indicates a structural origin of the higher activity. We tested this hypothesis by scaling the maximum ToFs from five subsequent measurements with the mean edge-length of the cubes and octahedra, respectively (Figure S18). This revealed very similar scaled activities for both particle types, which indicates that edge sites likely play a crucial role. Following this line, we can also explain the observed activity loss over time as the consequence of multiple exposures to 4.3 wt % ammonia and 4.3 wt % hydrogen peroxide in milli-Q water between each measurement series, since it is known that such treatment more efficiently etches edges and corners.^33−35^

**Figure 6:**
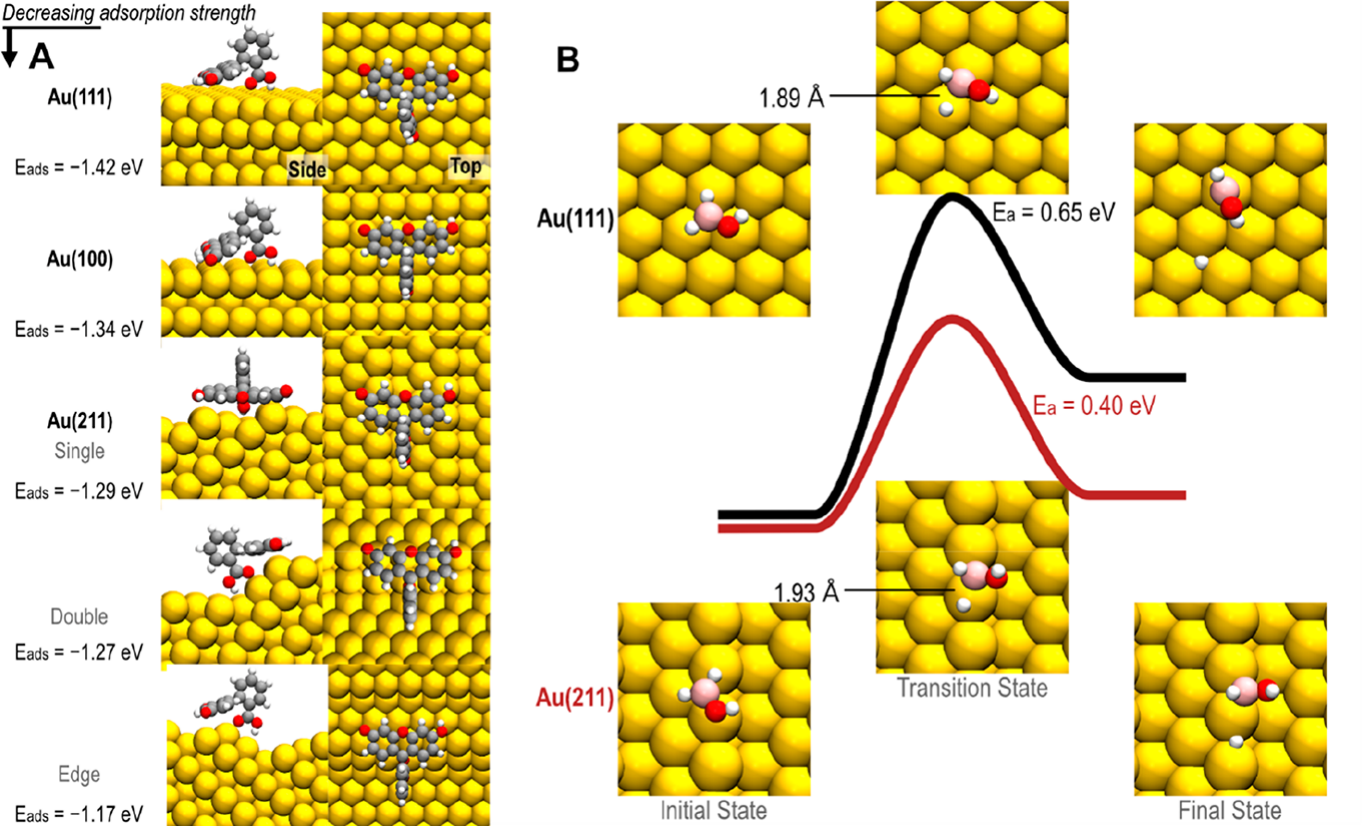
First-principles calculations of fluorescein adsorption energies and borohydride dehydrogenation activation barriers. (A) Adsorption energies, *E*_ads_, calculated from first-principles for reduced fluorescein adsorbed on flat Au(111) and Au(100) terraces together with surface models of steps and edges on nanoparticles. (B) The barrier for dehydrogenation of BH_2_OH on the flat Au(111) and stepped Au(211) surfaces, indicating structure sensitivity.

We resorted to first-principles calculations to corroborate the experimentally observed structure sensitivity and unravel its mechanistic origin. The shape of the Au colloidal crystals was modeled using flat Au(100) and Au(111) facets, as well as single-atom-height and multiatom-height stepped Au(211) surfaces, and finally, an extended 9 × 5 supercell was modified to model an edge on a nanoparticle, all together in order to mimic the curvature of the nanoparticle edges. These calculations show that the adsorption energy of reduced fluorescein decreases with increasing surface curvature because of deformations necessary in the fluorescein molecule to accommodate adsorption on a curved surface (Figure 6A). Further, we note that the adsorption energies of reduced fluorescein on the Au(100) and Au(111) facets are relatively high. This suggests that surface poisoning takes place at high fluorescein concentration and is caused by strong fluorescein adsorption, which is in good agreement with the experimentally observed catalyst deactivation at these conditions (compare with Figure 3), as well as surface coverage analysis using Langmuir–Hinshelwood reaction conditions (Supplementary Text Section 5 and Figure S19).

As a second aspect, we note that the ToF is determined by the slowest redox step since Rostamikia et al.^36^ showed that the dehydrogenation of BH_2_OH is rate-limiting on Au(111). Our first-principles results additionally show that the barrier associated with this dehydrogenation is structure sensitive, i.e., the barriers on flat Au(111) and stepped Au(211) are 0.65 and 0.40 eV, respectively (Figure 6B). This significantly lower value for the stepped surface corroborates our hypothesis that the abundance of edge sites dictates the ToF of the reaction on faceted Au nanocrystals.

Projecting these results onto the experimentally observed highest activity of octahedral particles, we propose the following mechanism. When decreasing the fluorescein concentration from the regime where the particle is completely poisoned due to strong fluorescein adsorption, it is eventually activated at the sites with the lowest fluorescein adsorption energy, where the poisoning is weakest. As our first-principles calculations have revealed, these sites are in regions with high surface curvature, i.e., at edges and corners. Hence, since the octahedra have the largest fraction of high-curvature edge sites of all considered particle types in our study (Figure 5C and Figures S5, S7), they are likely to be activated slightly more easily and, once activated, exhibit the highest max ToF thanks to the significantly lower barrier for the rate-limiting borohydride dehydrogenation, which is in excellent agreement with the experimental observations (Figure 5A,B and Figure S20).

## Conclusions

We have presented a nanofluidic device for trapping and counting individual colloidal nanocrystals at well-defined positions where they can be analyzed in a highly parallelized fashion with respect to their catalytic activity using fluorescence microscopy. Consecutive trapping of nanocrystals with different shapes in the same device enables the direct assessment of structure sensitivity at identical reaction conditions for all particles at the single nanoparticle level. Using the reduction of fluorescein by sodium borohydride as model reaction, we revealed distinct transitions from a surface poisoned, to a highly active, to a mass-transport limited reaction regime, together with a broad range of single-particle-specific catalytic activities for 32 Au faceted spheres because of their widely varying structures. Furthermore, the simultaneous assessment of the catalytic properties of Au faceted spheres, cubes, and octahedra revealed overall very similar behavior but with distinctly higher maximum ToFs for the octahedral crystals. Resorting to first-principles calculations, we revealed the origin of this behavior as the consequence of two different processes. First, the poisoning of the surface at high reactant concentrations due to strong adsorption of fluorescein is lifted earlier on surfaces with high curvature, such as steps and edges, thanks to their slightly lower fluorescein adsorption energies. Second, the activation barrier of the rate-limiting borohydride oxidation step is significantly reduced at highly curved edge sites, which explains the experimentally observed highest activity for Au octahedra. This highlights the importance of nanoparticle shape engineering in borohydride-mediated catalysis applied in, e.g., DBFCs or organic chemical synthesis, as well as the potential of nanofluidic devices for the study of structure–function correlations on individual colloidal nanocrystals. A future prospect, to fully realize the potential of this platform and enable structure function correlations with atomic-level precision, is to combine it with *in situ* electron microscopy characterization to, e.g., characterize particle faceting or defects before, during, and after reaction. As another future prospect the platform will, e.g., enable investigating the impact of surfactants that can be removed from, and reapplied to, trapped particles without inducing their aggregation.

## Methods

### Instruments

All fluorescence microscopy and dark-field scattering microscopy (DFSM) experiments were done using a Zeiss Axio Observer Z1 microscope with a Colibri 7 LED light source, an Andor iXon Ultra 888 EMCCD camera, and an alpha Plan-Apochromat 63×/1.46 Oil Corr M27 objective. A Hereus Multifuge X1 with a Fiberlite F1(5–8) × 50cy Fixed Angle Rotor (for volumes >5 mL) and a Mini spin with KL125 (9 cm) rotor (for volumes <5 mL) were used for the centrifugation of nanoparticles. UV–vis absorption spectra were measured at room temperature with an Agilent Cary-60 spectrophotometer equipped with a xenon flash lamp (80 Hz). Zeta-potential was measured with a Malvern Panalytical Zetasizer Nano ZS, while transmission electron microscopy (TEM) images and electron diffraction patterns were obtained on a FEI Tecnai T20 equipped with a LaB6 filament and Orius CCD and operated at 200 kV. A Disco DAD3350 Dicing saw was used for dicing of the nanofluidic devices. The SEM images of chips with removed lids were acquired with a SEM Zeiss Supra 55 after carbon coating with a AVAC HVC600 thermal evaporator.

### Chemical Reagents

Reagents for the reduction of fluorescein (fluorescein sodium salt, BioReagent, suitable for fluorescence) by borohydride (sodium borohydride, powder, ≥ 98.0%) were purchased from Sigma-Aldrich. For nanoparticle synthesis, Au(III) chloride trihydrate (HAuCl_4_·3H_2_O, ≥99.9%, Sigma-Aldrich), hexadecyltrimethylammonium chloride (CTAC, 99%, Acros Organics), sodium borohydride (NaBH_4_, ≥98%, Sigma-Aldrich), sodium bromide (NaBr, ≥99%, Sigma-Aldrich), potassium iodide (KI, ≥99%, Sigma-Aldrich), l-ascorbic acid (≥99%, Sigma-Aldrich), silver nitrate (AgNO_3_, ≥99.0%, Sigma-Aldrich), polyvinylpyrrolidione (PVP, average MW = ∼55 000, Sigma-Aldrich), sodium citrate tribasic dihydrate (Sigma-Aldrich, ≥99.0%), acetone (AnalaR NORMAPUR, VWR), and hydrogen peroxide (H_2_O_2_, 30% w/w in H_2_O, Sigma-Aldrich) were used without further purification. Ultrapure water (Milli-Q Advantage A10 water purification, Merck) was used for all nanoparticle synthesis and washing.

### Synthesis of Nanoparticles

Au nanocubes and nano-octahedra were synthesized using a seed-mediated growth method.^37^ Seeds were prepared in a 20 mL glass vial by reducing 10 mL of an aqueous solution of HAuCl_4_ (2.5 × 10^–4^ M) and CTAC (0.10 M) with 0.45 mL NaBH_4_ (0.02 M) while stirring vigorously (1000 rpm) at room temperature (19 °C). The resulting solution turned orange-brown immediately after the addition of NaBH_4_ and was stirred for 2 min before aging, without stirring, for 1 h in a 35 °C water bath. Further growth into nanocubes or nano-octahedra was induced in a two-step growth process in 20 mL glass vials. First, a growth solution was prepared in two vials for each synthesis. For nanocubes, these growth solutions consisted of 0.32 g of CTAC, 9.565 mL of water, 250 μL of HAuCl_4_ solution (0.01 M)_,_ and 10 μL of NaBr solution (0.02 M). For nano-octahedra, the growth solutions consisted of 0.32 g of CTAC, 9.495 mL of water, 250 μL of HAuCl_4_ solution (0.01 M), and 5 μL of KI solution (0.01 M). All growth solutions were immersed in a 35 °C water bath and stirred (500 rpm) until all CTAC was fully dissolved. Next, 150 or 220 μL of 0.04 M l-ascorbic acid (0.04 M) was added to the growth solutions for nanocubes or nano-octahedra, respectively, and the solutions changed color from light yellow to colorless. Finally, 25 μL (for nanocubes) or 30 μL (for nano-octahedra) of seed solution was added to the first vial (A) while stirring vigorously. After ∼5 s, as the color of solution changed to a light pink, the same volume (25 μL for nanocubes or 30 μL for nano-octahedra) of solution A was transferred to the second vial (B) and stirred for ∼10 s until a faint pink color just showed in the solution. Stirring was stopped, and the final solutions were left undisturbed for 15 min, during which time the nanocube solution turned a cloudy pink and the nano-octahedra solution changed to a dark purple. Nanoparticles were then centrifuged at 6000 rpm for 10 min and redispersed in water. This centrifugation was repeated to remove excess reactants, and the final solution was concentrated to a volume of 2.45 mL, which resulted in an Au concentration of ∼0.2 mg/mL.

Citrate-stabilized Au spheres (100 nm diameter, OD 1, stabilized suspension in citrate buffer) were purchased from Sigma-Aldrich.

### Ligand Exchange from CTAC to Mixed PVP and Citrate

A ligand exchange procedure was adapted from Zhou, et. al.^26^ in order to exchange the positively charged surfactant, CTAC, for the negatively charged polymer, PVP, and the negatively charged ligand, citrate. First, in a 20 mL glass vial at room temperature, 4.8 mL of a 0.047 M PVP solution (0.047 M) was mixed (500 rpm) with 1.0 mL of the final solution of CTAC-stabilized nanocubes or nano-octahedra described above and 0.1 mL of 0.04 M ascorbic acid (0.04 M). A 1.0 mL aliqout of a 3 × 10^–4^ M AgNO_3_ solution (3 × 10^–4^ M) was added to the solution and stirred for 10 min. At that time, the color changed to a red-orange color for the nanocubes and a bright pink for the nano-octahedra. Acetone was added in a 2:1 ratio to the nanoparticles, and then the solution was centrifuged at 5500 rpm for 30 min. The supernatant was removed, and the nanoparticles were redispersed in 200 μL of 0.001 M PVP + 0.01 M sodium citrate solution in a 4 mL glass vial. This solution was then immediately etched with 0.9 mL of 3% H_2_O_2_, while stirring at room temperature, for 3 h, at which point the solutions returned to their original colors (cloudy pink for nanocubes and dark purple for nano-octahedra). The nanoparticles were centrifuged at 13 400 rpm for 10 min, redispersed in 100 μL of 0.001 M PVP + 0.01 M sodium citrate solution, and allowed to sit for 16 h at room temperature. Then, 0.9 mL of water was added, and the nanoparticles were centrifuged again at 8500 rpm for 10 min and finally redispersed in 1 mL water.

### Fabrication of Nanochannel Traps

Vertical constrictions inside nanofluidic channels were fabricated by replacing step (e) in the subsection ‘Fabrication of nanochannels’ in the Supporting Information of our previous article^10^ with the following steps: (e1) Reactive-ion etching (RIE) for 5 s at 40 mTorr chamber pressure, 40 W RF-power, 40 sccm O_2_ flow (descum). RIE for 90 s at 20 mTorr chamber pressure, 50 W RF power, 200 W ICP power, 20 sccm O_2_ flow, 50 sccm Cl_2_ flow (selective Cr hard-mask etch). RIE for 40 s at 8 mTorr chamber pressure, 40 W RF power, 50 sccm NF_3_ flow (30 nm etch depth in thermal oxide). (e2) Spin coating maN2403 (Microchemicals) at 6000 rpm for 60 s, and soft baking (HP) at 90 °C for 2 min. (e3) Electron-beam exposure of one line of 1 μm width across the nanochannels at the position of the constrictions at 10 nA with a shot pitch of 10 nm and 700 μCcm^–2^ exposure dose. (e4) Development in maD525 for 90 s, rinsing in water, and drying under N_2_ stream. (e5) RIE for 5 s at 40 mTorr chamber pressure, 40 W RF power, 40 sccm O_2_ flow (descum). RIE for 160 s at 8 mTorr chamber pressure, 40 W RF power, 50 sccm NF_3_ flow (additional 120 nm etch depth in thermal oxide).

### PSQ Bonding and Scanning Electron Microscopy (SEM) Imaging

The process of polysilsesquioxane (PSQ) bonding is schematically shown in Figure S1. The 4 in. SiO_2_ wafer with micronanofluidic features and a 4 in. double-side-polished borosilicate glass (175 μm thickness, Si-Mat Germany) were piranha cleaned (3:1 concd H_2_SO_4_/H_2_O_2_, 150 °C) for 15 min. PSQ was freshly prepared before bonding by mixing Hardsil (AP grade, Gelest Inc.) with O-xylene (Fisher Scientific) in a 1:2 ratio. Then, PSQ was spin-coated (3000 rpm, 30 s) on piranha-cleaned borosilicate glass and cured at 220 °C for 30 min. The SiO_2_ wafer was first treated with O_2_ plasma using RIE (Plasmatherm BatchTop, sccm O_2_, 500 mT pressure, 100 W power, 1 min), and then the PSQ-coated side of the borosilicate glass wafer was O_2_ plasma treated (10 sccm O_2_, 250 mT pressure, 50 W power, 2 min), and both wafers were brought together to enable bonding. The bonded wafer was then diced into individual chips.

After the particles had been trapped, the chip was reopened using a tweezer to bend-off the lid starting at a chip edge (Figure S2). The chip was then coated with 3 nm carbon using a thermal evaporator before SEM imaging. The SEM images were recorded at 15 kV acceleration voltage and a working distance of 2 mm with an in-lens detector.

### Density Functional Theory Calculations

The calculations were performed using GPAW. The projector augmented wave method^38,39^ was used to model the interaction between the valence electrons and the core. The Kohn–Sham orbitals and the corresponding density were represented on a real-space grid with a spacing of 0.2 Å. The projector augmented wave method^40^ was used to model the interaction between the valence electrons and the core. Reciprocal space integration over the Brillouin zone was approximated with Fermi–Dirac distribution with a width of 0.1 eV. The exchange-correlation interaction was treated using the vdW-DF-cx functional,^41,42^ which includes van der Waals interactions into the exchange-correlation. Solvent effects were modeled using the continuum solvent model.^43^

In the case of fluorescein adsorption, the surface models consisted of flat terraces, i.e. Au(100) and Au(111), both with a width of 5 × 5 and four layers thick, and model surfaces for steps [here represented by single-atom-height step Au(211)] and edges [here represented by double-atom-height step Au(211)stepped surfaces, here represented by Au(211) and Au(211) with a double step] both with a width of 6 × 5 and five layers thick. Finally, we include an larger supercell, 9 × 5 Au(211) at six layers thick, in which we construct an edge by removing some of the layers. The bottom layer was kept fixed, and periodic surface slabs were separated by a vacuum of 26 Å. The reciprocal space was sampled using a Monkhorst–Pack grid of 4 × 4 × 1 for all of the surfaces, except for the edge surface model where the Monkhorst–Pack grid was 2 × 4 × 1. The free Fluorescein was modeled in a supercell of 24 Å × 23 Å × 20 Å, and the corresponding Brillouin zone was sampled using the Gamma-point. In the case of dehydrogenation of BH_2_OH, the surface model consisted of Au(111) with a width of 3 × 3 and four layers thick and Au(211) with a width of 3 × 2 and five layers thick. The bottom layer was kept fixed, and periodic surface slabs were separated by a vacuum of 26 Å. The reciprocal space was sampled using a Monkhorst–Pack grid of 6 × 6 × 1.

The system was considered to be relaxed when the largest atomic force in the system was smaller than 0.03 eV/Å. The adsorption energy was calculated in reference to the bare Au surface and the separate fluorescein, whereas in the case of BH_2_OH the reference was the adsorption on Au(111). In both cases, the energies included solvent effects. The transition states (TSs) were obtained using the climbing NEB method.^44,45^

## Supporting Information Available

Video S1: Video obtained with DFSM when trapping 100 nm Au spheres in the nanofluidic channels with vertical constrictions (MP4)
Figures S1–S20, Table S1, and references (46–50) (PDF)

## Notes

The data and code that support the findings of this study are available from the corresponding authors upon request.

## ABBREVIATIONS

DBFC: direct borohydride fuel cell
ToF: turnover frequency
SEM: scanning electron microscopy
PSQ: polysilsesquioxane
CTAC: hexadecyltrimethylammonium chloride
DFSM: dark-field scattering microscopy
TEM: transmission electron microscopy
