# Supplementary material for: Nanofluidic Trapping of Faceted Colloidal Nanocrystals for Parallel Single-Particle Catalysis
Source: ACS Nano. 2022 Sep 2;16(9):15206–14. doi: 10.1021/acsnano.2c06505 (PMC9527799; doi:10.1021/acsnano.2c06505)
Supplement: Supplementary file 2 — nn2c06505_si_002.pdf [file nn2c06505_si_002.pdf]

Supplementary Information

Nanofluidic Trapping of Faceted Colloidal Nanocrystals for Parallel Single Particle Catalysis

Sune Levin[1], *Sarah Lerch*[2], *Astrid Boje*[3], *Joachim Fritzsche*[3], *Sriram KK*[1], *Henrik Ström*[4,5], *Kasper Moth-Poulsen*[2,6,7], *Henrik Sundén*[2,8], *Anders Hellman*[3,9], *Fredrik Westerlund*[1]* *and Christoph Langhammer*[3]*

[1]Department of Biology and Biological Engineering, Chalmers University of Technology; SE-412 96 Gothenburg, Sweden

[2]Department of Chemistry and Chemical Engineering, Chalmers University of Technology; SE-412 96 Gothenburg, Sweden

[3]Department of Physics, Chalmers University of Technology; SE-412 96 Gothenburg, Sweden

[4]Department of Mechanics and Maritime Sciences, Chalmers University of Technology; SE-412 96 Gothenburg, Sweden

[5]Department of Energy and Process Engineering, Norwegian University of Science and Technology; NO-7034 Trondheim, Norway

[6]Institute of Materials Science of Barcelona, ICMAB-CSIC; ES-08193 Bellaterra, Barcelona, Spain

[7]Catalan Institution for Research and Advanced Studies, ICREA; ES-08010 Barcelona, Spain

[8]Department of Chemistry & Molecular Biology, University of Gothenburg; SE-412 96 Gothenburg, Sweden

[9]Competence Centre for Catalysis, Chalmers University of Technology; SE-412 96 Gothenburg, Sweden

*Corresponding authors. Email: fredrikw@chalmers.se; clangham@chalmers.se

This document includes:

Supplementary Text

Figs. S1 to S20

Table S1

Caption for Video S1

Other Supplementary material for this manuscript include the following:

Video S1

Supplementary Text

Section 1: Particle trapping in chip with PSQ-bonded lid

PSQ is a polymer with siloxane bonds (Si-O-Si) and is known for its excellent aberration resistance which is why it is widely used in coating applications(*23, 46*). PSQ is highly transparent and has a high mechanical modulus. The fact that its chemical structure [RxSiOy]n (where R is a hydrocarbon group) is similar to that of polydimethylsiloxane (PDMS) makes it suitable for micro- and nanofluidic bonding. Gu et.al, established a PSQ bonding process suitable for nanochannels as small as 8 nm in height and this method has further been used in various applications involving micro- and nanofluidic device fabrication (**Fig. S1**)(*24, 47-49*). Here, we use PSQ-bonding of the transparent lids on our nanofluidic chips to enable lid-removal after trapping of colloidal Au nanoparticles to enable SEM imaging of the traps. The process flow of the PSQ-bonding used is depicted in **Fig. S1** and the correspondingly obtained result in **Fig. S2**. SEM images taken from a large number of particle traps are depicted in **Fig. S3**. **Fig. S4** then summarizes the comparison of trapping analyzed by SEM and DFSM.

Section 2: Measurement and interpretation of fluorescence intensity data

Measurements of turnover frequencies (ToFs) were executed with an epi-fluorescence microscope. During measurements, reactant concentrations of 18-40 μM fluorescein and 50 mM sodium borohydride in water were flushed through the nanochannel array with trapped particles, at a constant rate of 495 μm/s for the 32 Au spheres (**Fig. 3**) or 272 μm/s for the mixed particles (**Fig. 4**, **5**), because of slightly different nanofluidic chip design. The first design contained 3 sets of 100 nanochannels with different traps (vertical constriction, well or horizontal constriction) while the second design contained 3 sets of 100 nanochannels with the preferred trap type used in this work (vertical constriction). The measurements were only done in the nanochannels with the vertical constriction and the difference in flow speed is attributed to the total difference in pressure drop across all nanochannels between the two different chip designs. The flow speed was measured separately for each design by sequentially flushing fluorescein (100 μM) and water through the nanochannels at a flow pressure of 2000 mbar and by evaluating the velocity of the change in brightness. The change in brightness was evaluated by assigning a brightness cutoff which determines if each pixel in the channel is bright or not. The flow speed could then be derived from the change in number of bright pixels between each given frame. By evaluating the speed in both flow directions and taking the mean value from both directions, a flow speed without dependence of brightness cutoff was found (this procedure is also described in our previous study(*10*)). To verify that the interaction between particle and trap was strong enough to keep the particle in place during the subsequent catalysis experiment, we carried out a "stress test" for each chip by applying a reversed flow of milli-Q water for 20 minutes, while still imaging in DFSM mode. In this way, the eventual detachment of particles was easily detected, enabling the exclusion of corresponding channels from further analysis. During the reversed flow, the geometric structure of the trap "shields" the trapped particles from the flow since they are situated in the recirculation region behind the barrier. In other words, most of the flow through the constriction passes over the particles and thus affects them to much smaller extent than if the constriction would not have been there.

The fluorescein concentration decreased slowly during the experiments from the initial concentration down to 0, as a consequence of a slow background reaction occurring on unspecific sites provided by the large surface area of the microchannels before reaching the nanochannels (by the same principle as in our previous study(*10*)). To evaluate the intensity downstream of

the trapped particles, intensities from two types of reference channels (I_1 and I_2) were used. I_1 was recorded in channels that contained multiple nanoparticles and in which, for this reason, the reaction was always in the mass-transport-limited regime throughout the experiments. I_2 was recorded in channels without particles, which therefore showed no activity. Based on these reference intensities and the intensity in each channel (I_{chan}), the normalized intensity (I_{norm}) for each channel was calculated according to $I_{norm} = \frac{I_{chan}-I_1}{I_2-I_1}$. I_{norm} then corresponded to the fraction of non-reduced fluorescein molecules downstream of the nanoparticle for each channel. ToFs were then determined using equation: $ToF = (1 - I_{norm}) \cdot C \cdot V_{flow} \cdot N_{sites}$, where C is the incoming fluorescein concentration, V_{flow} is the volume flowing past the particle per second, and N_{sites} is the estimated number of surface atoms on the trapped particle in the corresponding nanochannel. For the colloidal nanoparticles N_{sites} was estimated from the particle mean characteristic length, l, (diameter for spheres and side lengths for cube and octahedra) measured with TEM (**Fig. S5**, **S7**). For the different shapes, surface areas were calculated according to $SA_{sphere} = l_{sphere}^2 \cdot \pi$, $SA_{cube} = l_{cube}^2 \cdot 6$, and $SA_{octahedra} = l_{octahedra}^2 \cdot 2\sqrt{3}$ for spheres, cubes and octahedra respectively. The surface areas were then multiplied with the packing factor, divided by the unit cell area, (f_p) of the corresponding predominant surface facets (100 for cubes (f_p = 12.1 nm^{-2}), 111 for octahedra(f_p = 13.9 nm^{-2}), and an equal mix of 100, 111 and 211 for spheres (f_p = 11.5 nm^{-2})) to arrive at the estimated number of surface atoms $N_{sites} = SA \cdot f_p$. For the calculation of edge sites (**Fig. 5C**), the total edge lengths ($l_{cube/octahedra} \cdot 12$) were divided by the distance between atoms along the edges (0.407 nm for cubes and 0.288 nm for octahedra). Note that since the edge length scales to the power of 1 with side length and the surface area scales with the power of 2, the fraction of edge sites is lower for longer side lengths.

Section 3: Day-to-day variations

A general complication in our experiments, and in experimental catalysis in general, is that conditions might vary slightly between measurements, which makes day-to-day experiments difficult to compare directly in a quantitative fashion. To exemplify this, **Fig. S10A** shows the maximum ToFs for the measurement series in **Fig. 3B**, while **Fig. S10B** displays maximum ToFs measured for the exact same 32 particles on three consecutive days, at nominally identical reaction conditions. The mean maximum ToF here varies between 0.2 and 0.9 s^{-1} for the different experiments. At the same time, the relative activity between the particles was clearly retained between experiments (**Fig. S10C**, indicating that the particles themselves remained essentially unchanged. This highlights the importance of ensuring identical reaction conditions and simultaneous measurements on differently shaped particles if subtle structure-related effects are to be discovered.

Section 4: Size from dark-field scattering intensity

To elucidate a potential influence of particle size on the measured activity traces, we examined the dark-field scattering intensity traces obtained during the particle trapping step (**Fig. 2**). Specifically, by assuming that the light scattering intensity of each particle, *I*, is proportional to the particle radius, *r*, as $I \propto r^6$, a particle size distribution could be derived from these experiments(*50*). Normalizing it with the mean particle size obtained from TEM images of particles from the same batch (**Fig. S5**) revealed a very similar size distribution (**Fig. S12A**), which meant that we could use the scattering signatures of the individual particles to estimate their size. To emulate a spread in nanoparticle sizes (x-axis in **Fig. 5A**), the characteristic length l

for each particle was estimated according to $l = (\frac{I_{scat}}{I_{mean}})^{\frac{1}{6}} \cdot l_{mean}$, where I_{scat} is the intensity step determined with DSFM of the corresponding particle, I_{mean} is the mean value of all intensity steps and l_{mean} is mean value of the characteristic length of the particle batch determined from TEM (**Fig. S12**). The surface area for each particle was then calculated from their individually estimated characteristic length the same way as described in the previous section (**Section 2**).

Section 5: Simulation of the reaction mechanism with Langmuir-Hinshelwood conditions

As the starting point for simulating the reduction of fluorescein as a Langmuir-Hinshelwood reaction, we adopted the same 1D model system that we used to simulate reactivity in a nanochannel in our previous study(10). This system accounts for several 1D channels, each one decorated with four nanoparticles with respective surface area 1 to 4, 16 and 128 (presented as the relative surface area, without unit, since the absolute value is not relevant when we later introduce arbitrary rate constants), and numerically iterates reaction rate, flow and diffusion until a steady state is reached. The reaction properties were then modified to be based on surface coverage (θ) with Langmuir-Hinshelwood conditions defined as

$$\frac{\partial \theta_A}{\partial t} = r_1 - r_3 = k_1^+ \cdot P_A \cdot \theta_* - k_1^- \cdot \theta_A - k_3^+ \cdot \theta_A \cdot \theta_B + k_3^- \cdot \theta_{AB} \cdot \theta_*$$
$$\frac{\partial \theta_B}{\partial t} = r_2 - r_3 = k_2^+ \cdot P_B \cdot \theta_* - k_2^- \cdot \theta_B - k_3^+ \cdot \theta_A \cdot \theta_B + k_3^- \cdot \theta_{AB} \cdot \theta_*$$
$$\frac{\partial \theta_{AB}}{\partial t} = r_3 - r_4 = k_3^+ \cdot \theta_A \cdot \theta_B - k_3^- \cdot \theta_{AB} \cdot \theta_* - k_4^+ \cdot \theta_{AB} + k_4^- \cdot P_{AB} \cdot \theta_*$$
$$\frac{\partial \theta_*}{\partial t} = -\frac{\partial \theta_A}{\partial t} - \frac{\partial \theta_B}{\partial t} - \frac{\partial \theta_{AB}}{\partial t}$$
$$r = k_3^+ \cdot K_1 \cdot K_2 \cdot P_A \cdot P_B (1 - \frac{P_{AB}}{K_1 \cdot K_2 \cdot K_3 \cdot K_4 \cdot P_A \cdot P_B}) \cdot \theta_*^2,$$

where θ_X are the different surface coverages or free sites (θ_*), r_i are the adsorption rate of fluorescein, the adsorption rate of borohydride, the rate of reaction and the desorption rate of the product, respectively, P_X is the concentrations of the different reactants and product, $k_i^{\pm}$ are rate constants, K_i are equilibrium constants, and A, B and AB represent fluorescein, borohydride, and reduced fluorescein, respectively. Initially, all rate constants ($k_i^{\pm}$) were assigned to 1. The rate constants of the reaction between borohydride and fluorescein were then increased to $k_3^+ = 100$ and $k_3^- = 10$ to ensure that all fluorescein is consumed in the channels with the largest particles (since that is what we observe experimentally for channels with multiple particles, i.e. larger surface area). Each rate constant was then varied one at a time to explore the effect on the ToF vs. incoming fluorescein concentration, and the conditions at which fluorescein poisoning occurs with a drastic decrease in ToF after reaching max ToF (compare **Fig. 3B** and **Fig. 4B** with **Fig. S19**). As the key result, we then observe a drastic decrease in activity for higher fluorescein concentrations only for low values of either k_1^- or k_4^-. This clearly indicates that the low activity for high concentrations of fluorescein is likely to be due to surface poisoning by the fluorescein and/or the reduced fluorescein bound to the catalyst surface. A lower value for k_1^- (**Fig. S19B-C**) results in a higher absorption which increases the activity at lower fluorescein concentrations but lowers the activity at higher fluorescein concentrations due to surface poisoning. A lower value of k_4^- (**Fig. S19D**) has a comparable effect, but mainly the activity at lower fluorescein concentrations is increased since more space is allowed for the

fluorescein to bind and the probability of the back reaction is decreased. When both values are lowered, a cooperative effect is observed (**Fig. S19E-F**). All in all, these simulations corroborate our hypothesis that the decreased activity at higher fluorescein concentration is caused by strong binding (i.e., low desorption rate) of fluorescein to the Au surface and in turn that the poisoning is likely lifted earlier if sites with lower adsorption energy (such as edges) are present.

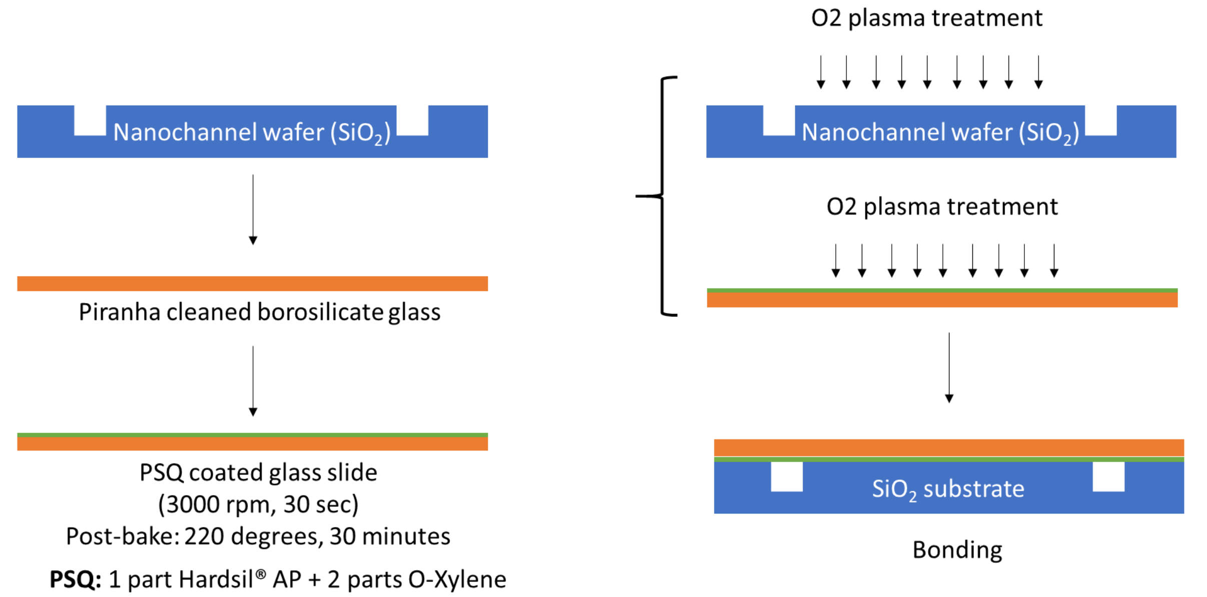

Fig. S1. *Schematic of the process flow during PSQ-bonding of a transparent lid onto a nanofluidic chip.*

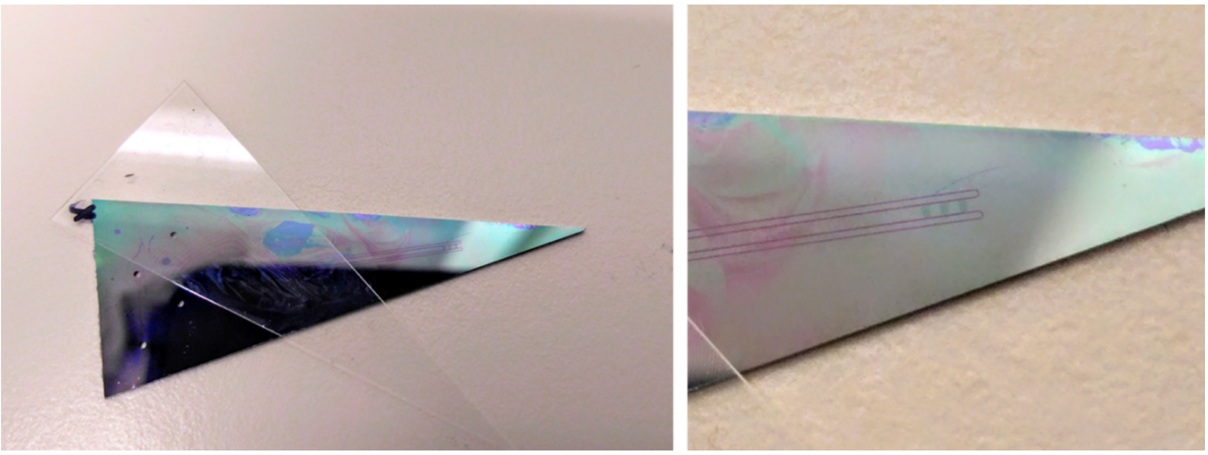

Fig. S2. *Photographs of a nanofluidic chip after particle trapping and lid un-bonding.*

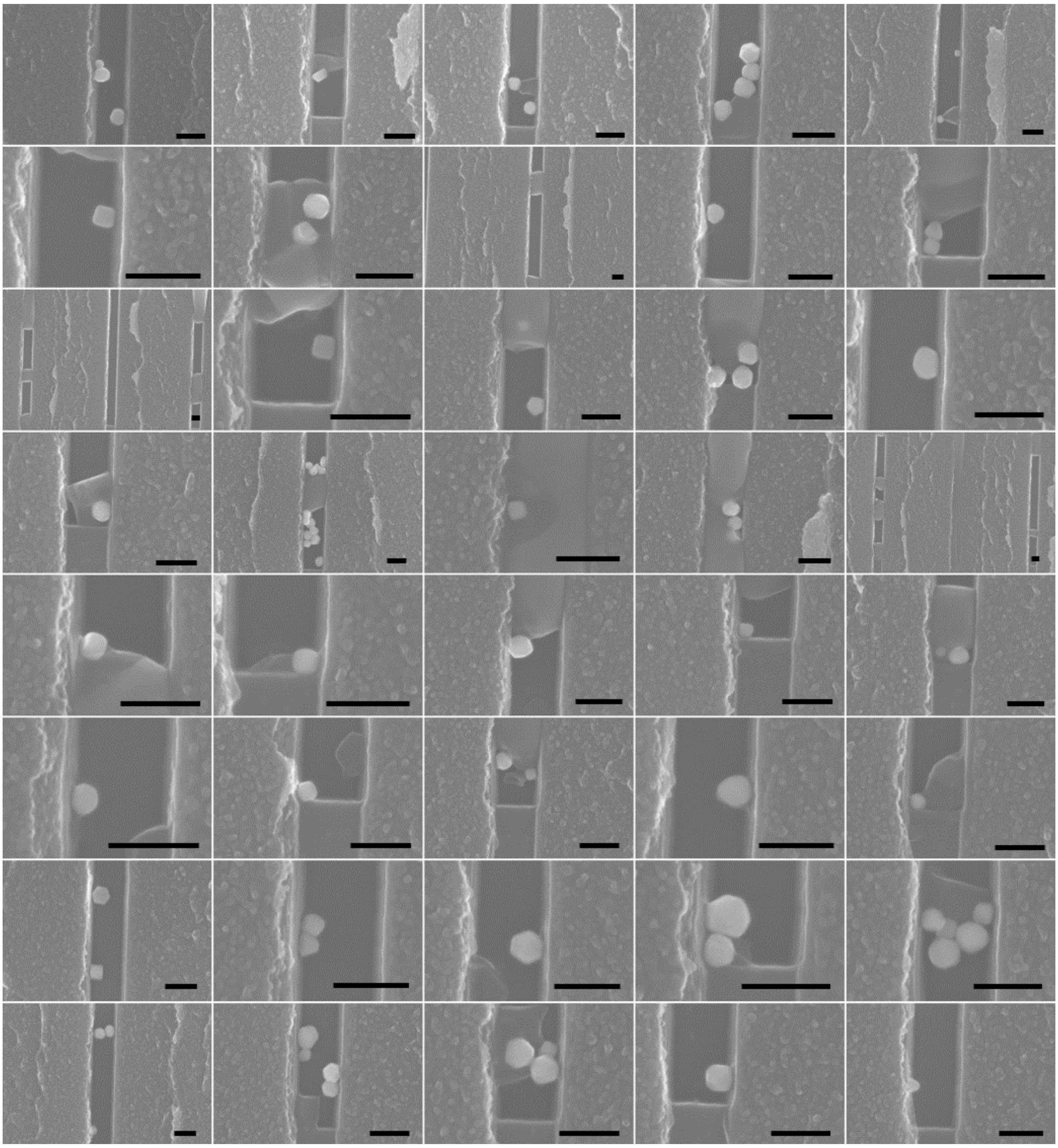

Fig. S3. *SEM images of all particle traps analyzed using the PSQ-bonded chip. The images are presented in the same order as the corresponding nanochannels appear in* ***Fig. S4*** *below. Scale bars are 200 nm.*

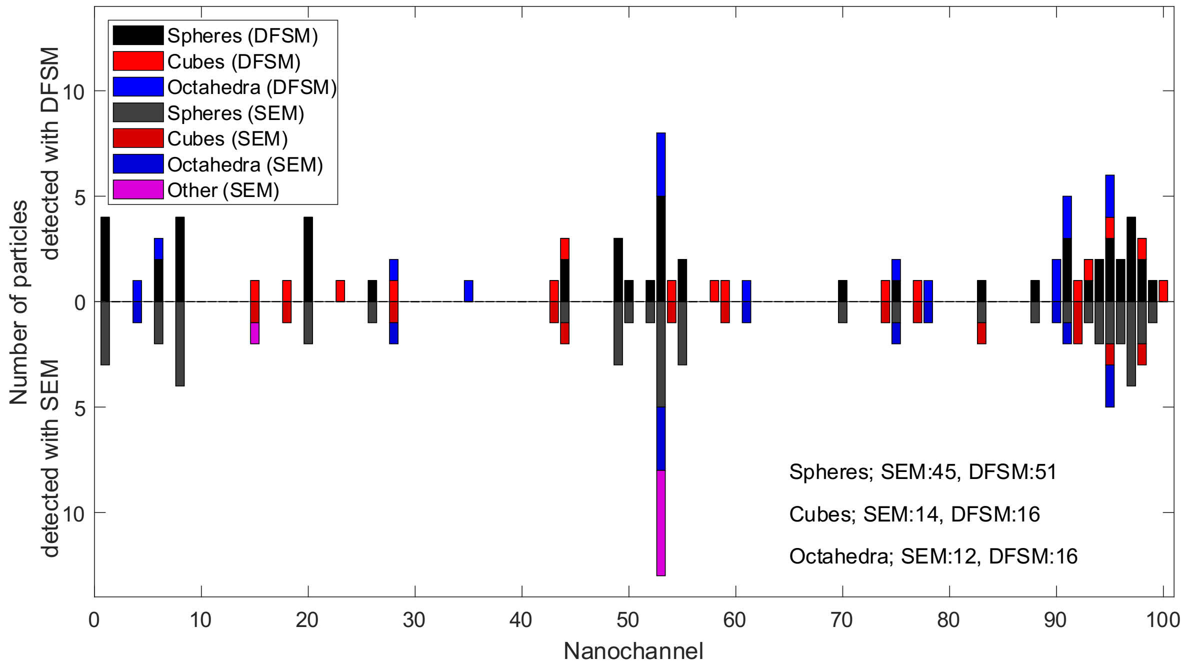

Fig. S4. *Comparison of number of counted particles in a chip when using online DFSM or post mortem SEM after removal of the lid from the same chip. In a few channels, additional particles were found in with SEM compared to DFSM. These can be attributed to particles that attached a few micrometers before reaching the trap (channel 15 and 53) or particles entering as dimers (channel 55 and 92).*

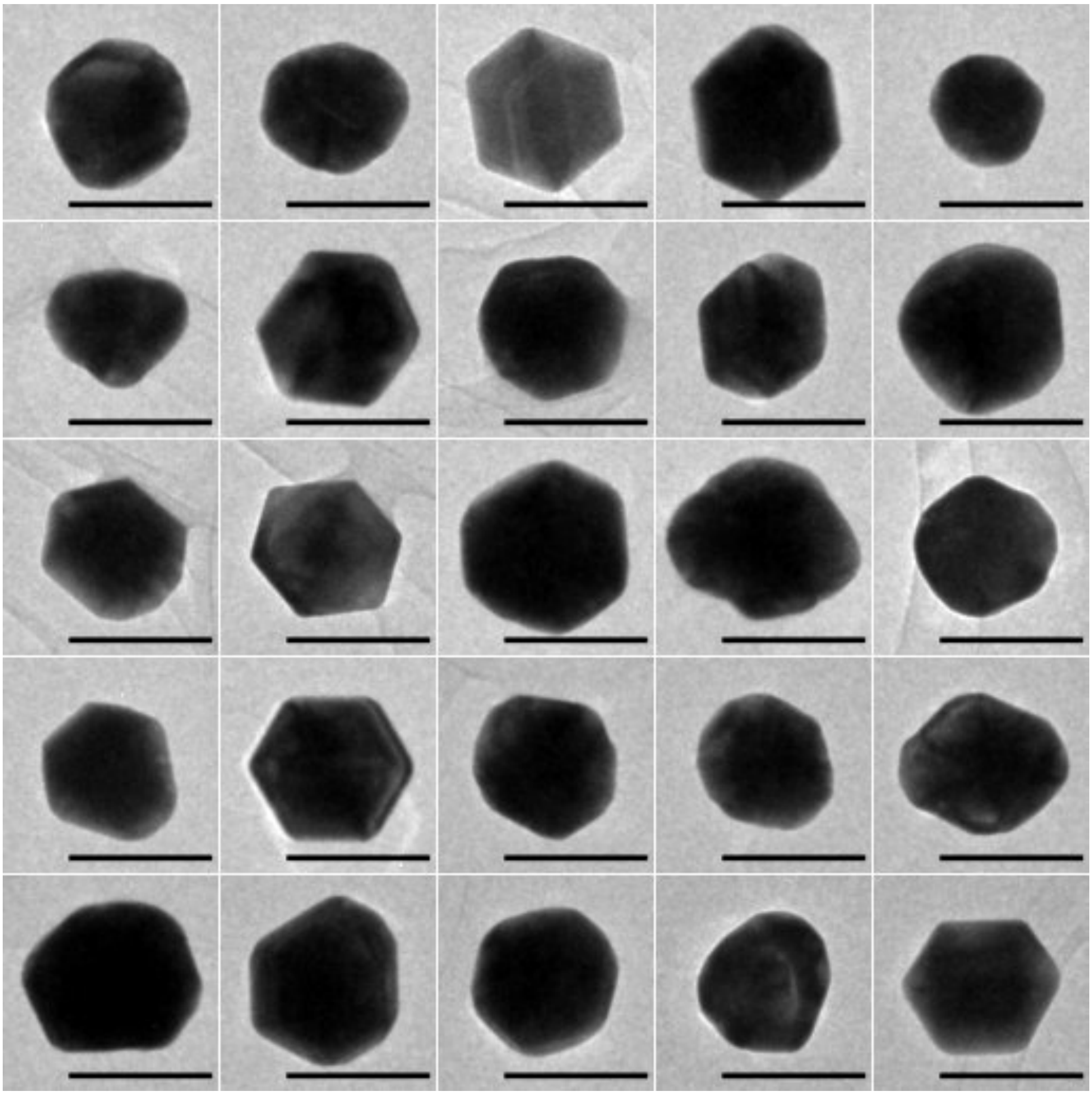

Fig. S5. *TEM images of Au faceted spheres representing the wide distribution of shapes and faceting in this particle population. Scale bars are 100 nm.*

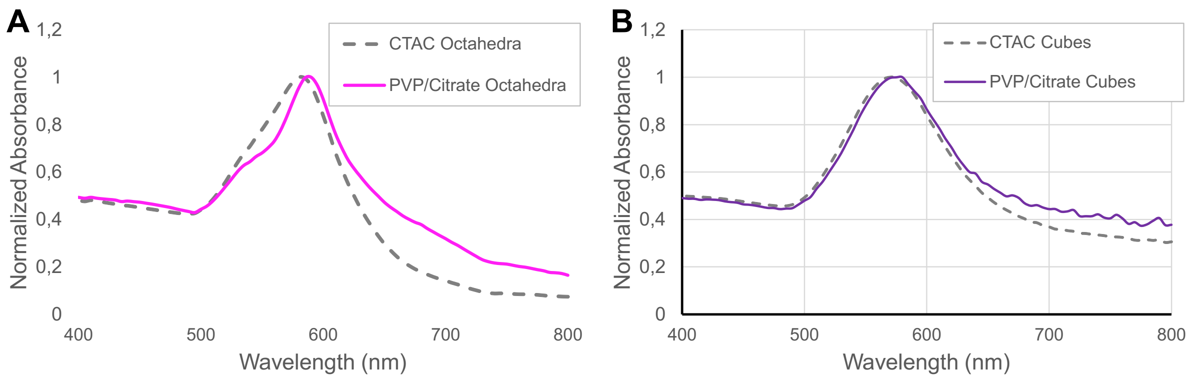

Fig. S6. *UV-vis absorption spectra for colloidally synthesized nanoparticles. (**A**) Octahedra stabilized with CTAC (gray, dashed line) before ligand exchange and with PVP/citrate (pink, solid line) after ligand exchange. Peak wavelengths are 580 nm and 590 nm, respectively. (**B**) Cubes stabilized with CTAC (gray, dashed line) before ligand exchange and with PVP/citrate (purple, solid line) after ligand exchange. Peak wavelengths are 570 nm and 580 nm, respectively. Slight red shift in both samples after ligand exchange is due to different refractive indices of CTAC and PVP/citrate mixture.*

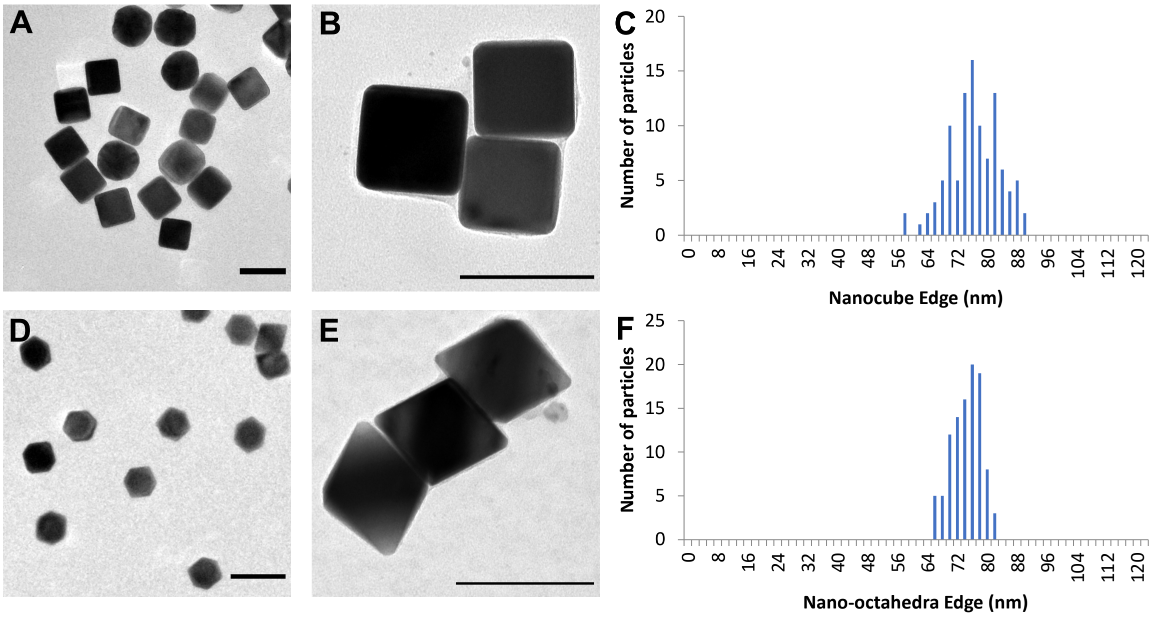

Fig. S7. *TEM images and size distributions for synthesized nanoparticles. (**A**-**B**) TEM images of cubes, with side length 75.5 ± 6.8 nm. (**C**) Size distribution of cubes, which represented 80% of the particles in solution (106 particles measured). **(D-E)** TEM images of octahedra, with side length 73.5 ± 3.9 nm. (**F**) Size distribution of octahedra, which represented 74% of the particles in solution (141 particles measured). All scale bars 100 nm.*

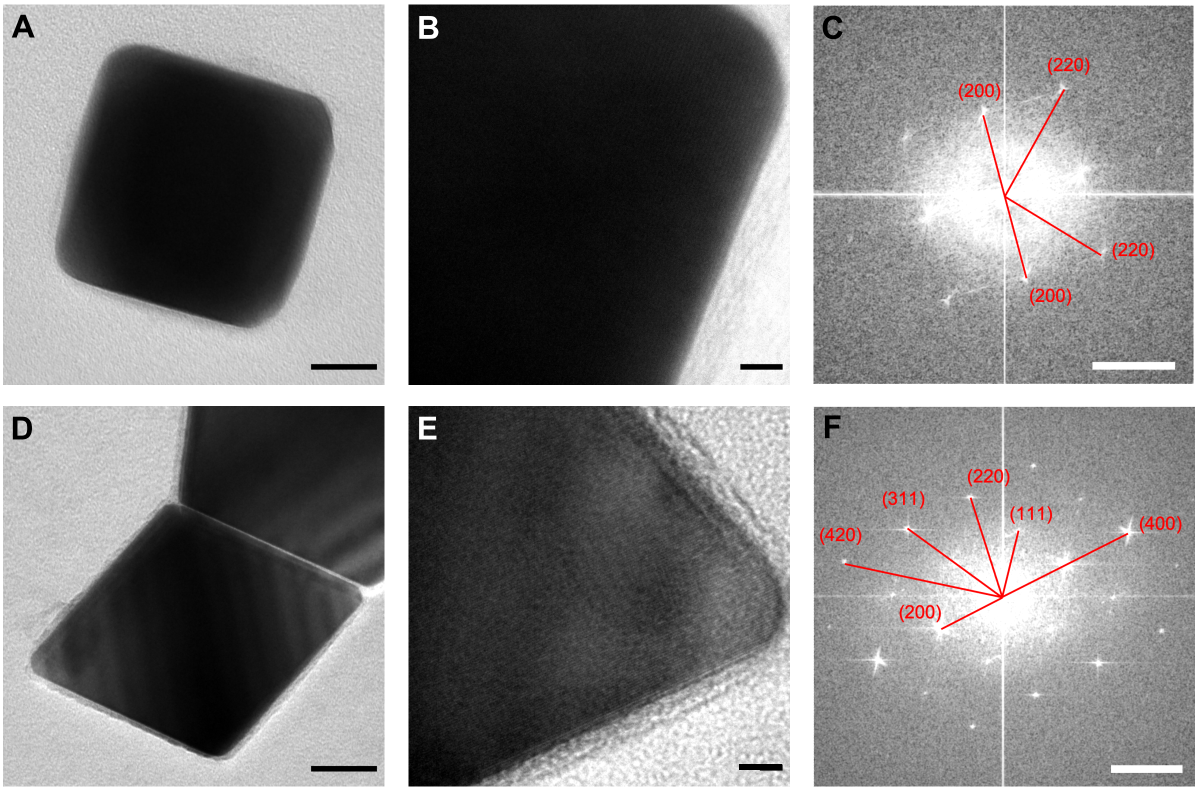

Fig. S8. (**A**) TEM image of Au cube. Scale bar 20 nm. (**B**) High resolution dark-field TEM image of the cube in (A). Scale bar 2 nm. (**C**) Corresponding selected area electron diffraction (SAED) pattern for the cube depicted in (A) & (B). Scale bar 5 nm^{-1}. Diffraction spots correspond to lattice parameters of 1.99 ± 0.05 Å and 1.42 ± 0.05 Å, matching the (200) and (220) planes for Au, confirming the presence of (100) surface facets characteristic for a cube. (**D**) TEM image of two Au octahedra. Scale bar 20 nm. (**E**) High resolution TEM image of one of the octahedra in (D). Scale bar 2 nm. (**F**) Corresponding SAED pattern. Scale bar 5 nm^{-1}. Diffraction spots correspond to lattice parameters of 2.32 ± 0.05 Å, 2.03 ± 0.05 Å, 1.40 ± 0.05 Å, 1.24 ± 0.05 Å, 0.99 ± 0.03 Å and 0.88 ± 0.05 Å, matching the (111), (200), (220), (311), (400) and (420) planes for Au, thereby confirming the (111) surface facets characteristic for an octahedron.

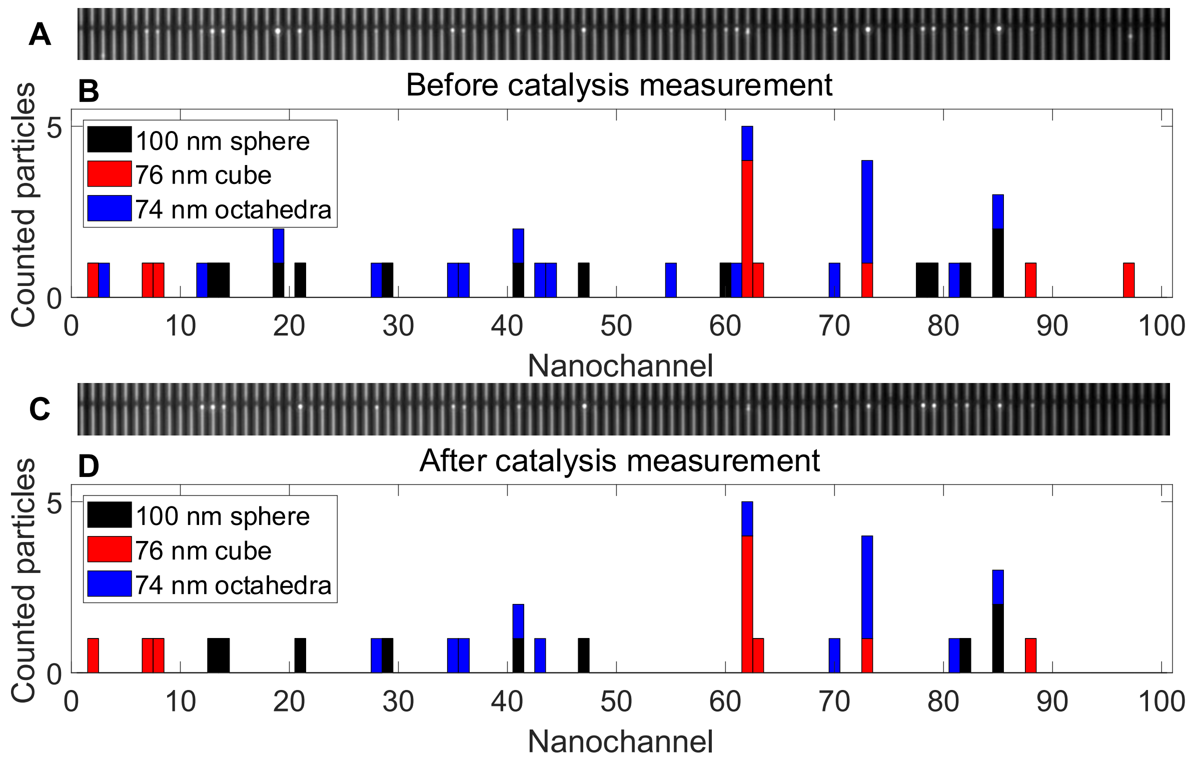

Fig. S9. *DFSM images (**A**, **C**) and corresponding extracted particle distributions in the chip directly after the consecutive trapping of spheres, cubes and octahedra (**B**) and after subsequent catalysis measurements (**D**).*

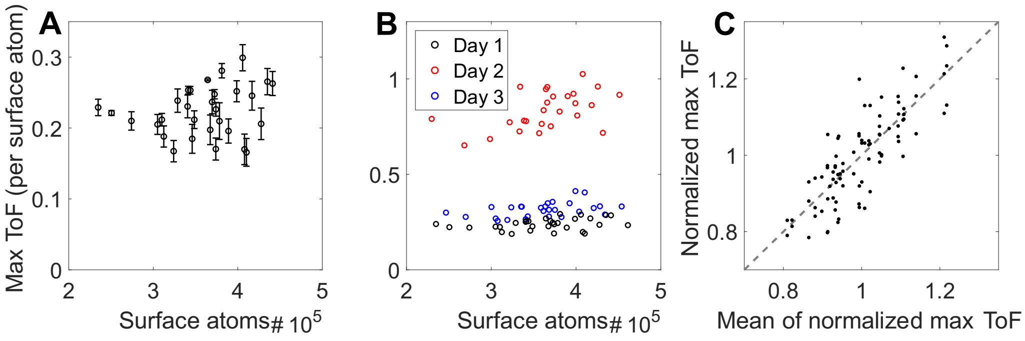

Fig. S10. *Demonstration of day-to-day variations seen between different experiments on the same particles using nominally identical reaction conditions. (**A**) Max ToF per surface atom for each of the 32 particles. Error bars show the standard deviation between the 4 measurements in **Fig. 3B**. (**B**) Max ToF from measurements done on different days displaying variation in determined max ToF. (**C**) Normalized max ToF as a function of mean value for the three measurements done on different days. The correlation illustrates that the particles retain their corresponding activity relative to the other particles and that the day-to-day variations thus only affect the extracted absolute ToF values and that to the same extent for all particles.*

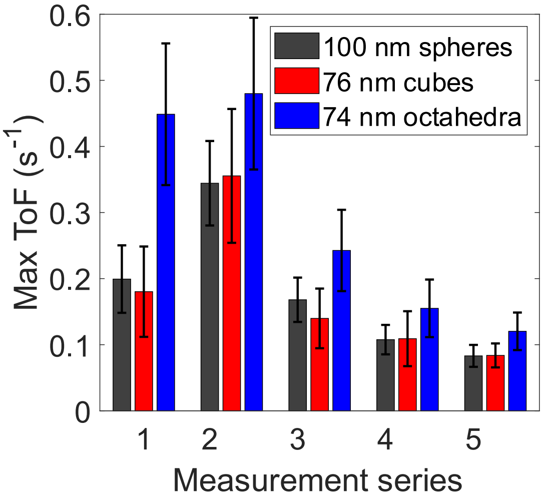

Fig. S11. *Average maximal ToF for each particle type across 5 measurement series. Error bars display the standard deviation within each particle type for each series.*

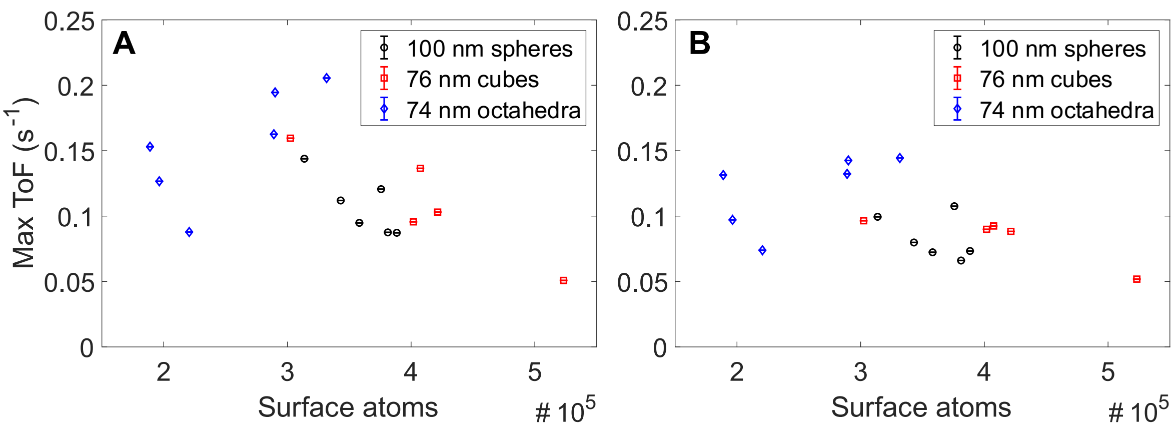

Fig. S12. *Maximum ToFs for all particles in measurement series 4 (**A**) and 5 (**B**). While the overall activity has decreased (compare with **Fig. 5**) the relative activity between individual particles is to a large extent preserved.*

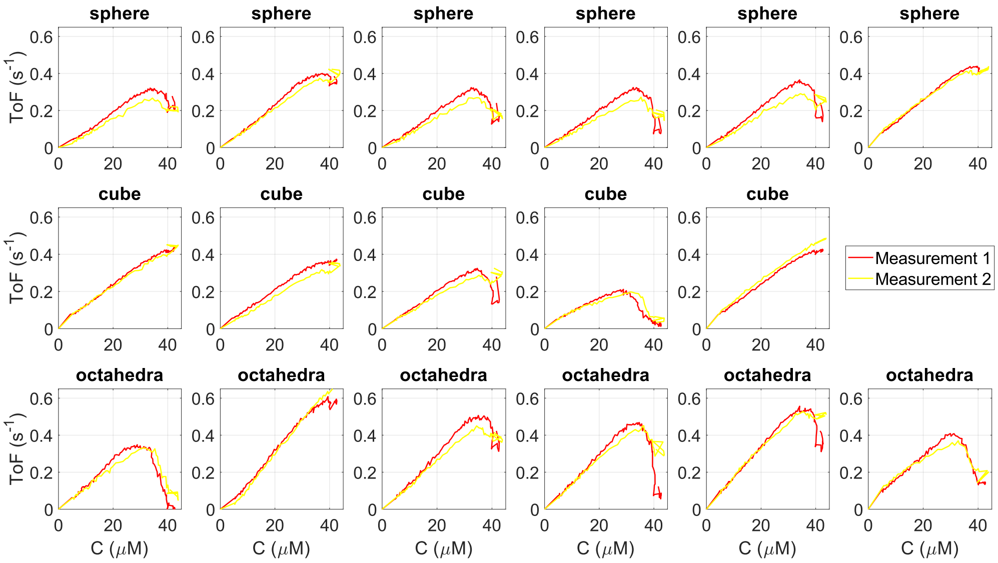

Fig. S13. *The second measurement series of fluorescein reduction with sodium borohydride on individual Au faceted spheres, cubes and octahedra. The ToF per surface atom is measured simultaneously in the same chip upon two subsequent fluorescein concentration sweeps starting at* C_{start} *= 40 μM.*

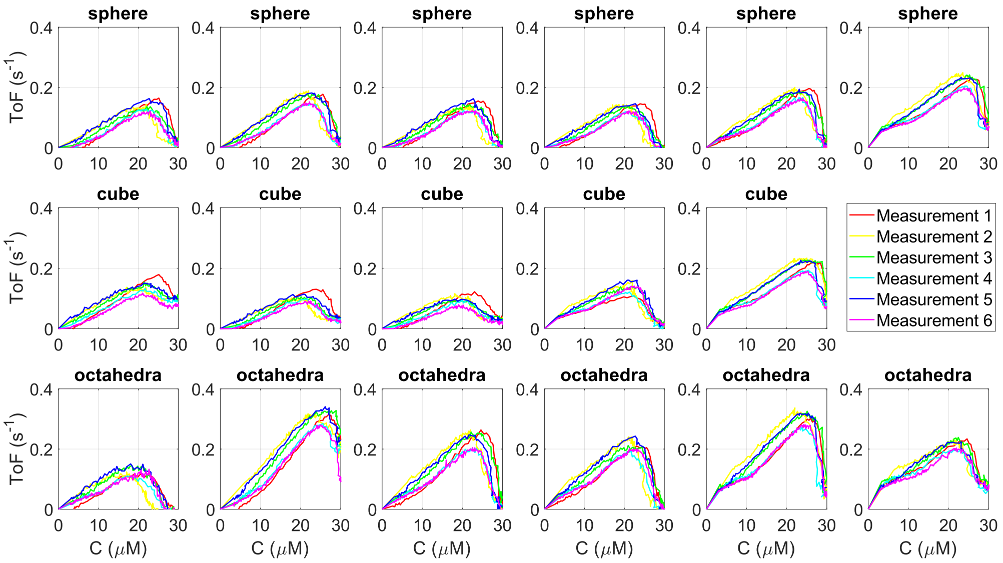

Fig. S14. *The third measurement series of fluorescein reduction with sodium borohydride on individual Au faceted spheres, cubes and octahedra. The ToF per surface atom is measured simultaneously in the same chip upon six subsequent fluorescein concentration sweeps starting at* $C_{start} = 30\ \mu M$.

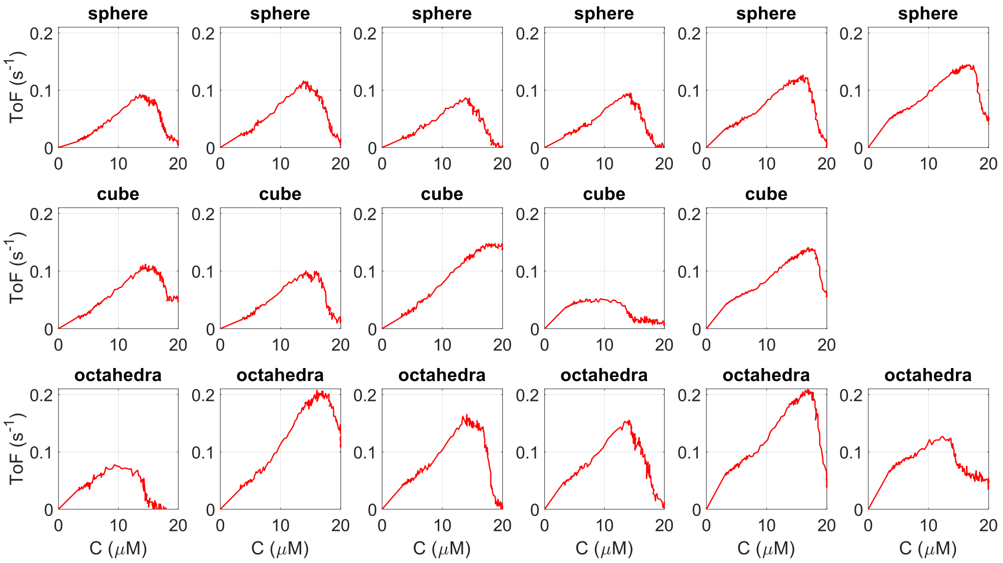

Fig. S15. *The fourth measurement series of fluorescein reduction with sodium borohydride on individual Au faceted spheres, cubes and octahedra. The ToF per surface atom is measured simultaneously in the same chip upon a fluorescein concentration sweep starting at C_{start} = 20 μM.*

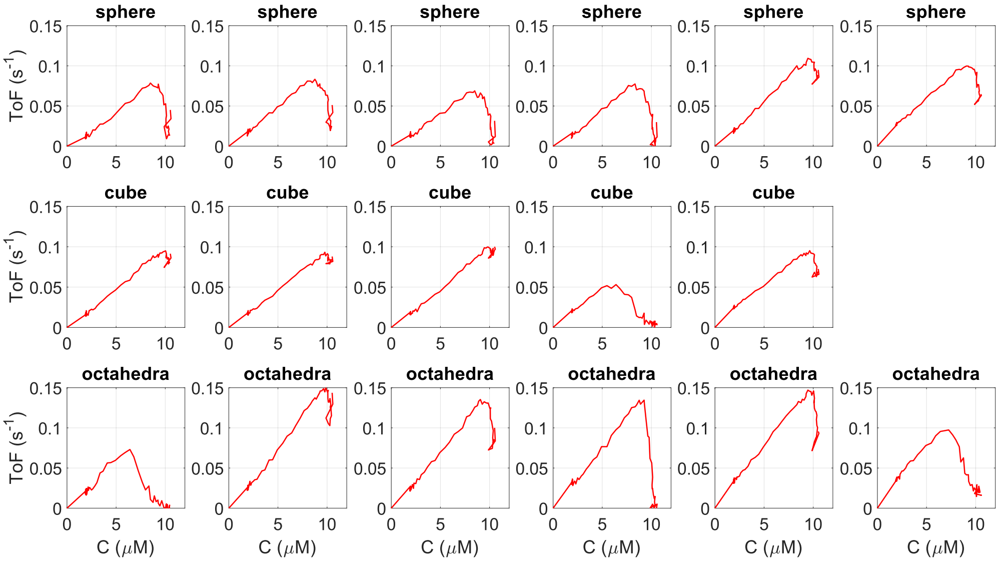

Fig. S16. *The fifth measurement series of fluorescein reduction with sodium borohydride on individual Au faceted spheres, cubes and octahedra. The ToF per surface atom is measured simultaneously in the same chip upon a fluorescein concentration sweep starting at C_{start} = 10 μM.*

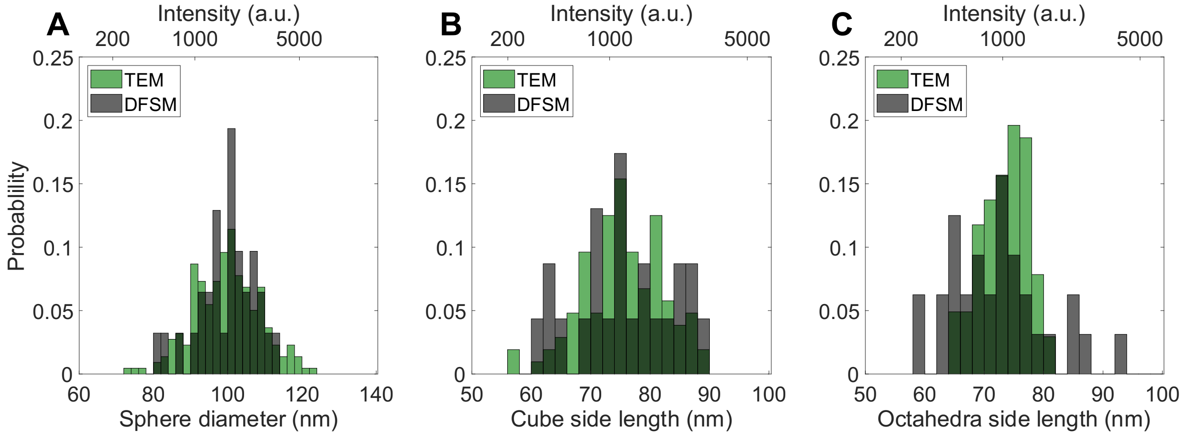

Fig. S17. *Particle size histograms, as obtained from TEM compared to characteristic length determined from intensity steps during particle trapping for (**A**) spheres, (**B**) cubes and (**C**) octahedra. Note the very good agreement.*

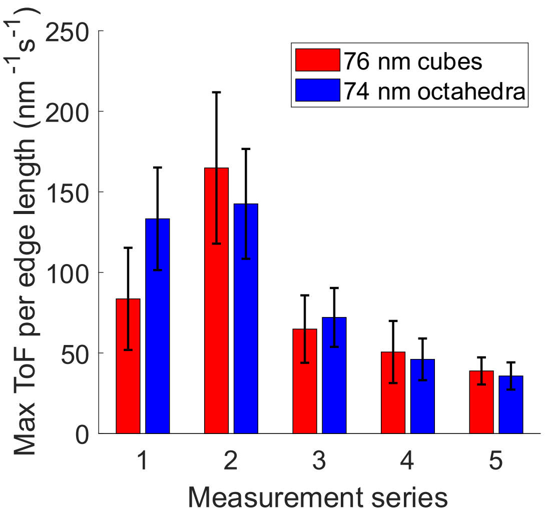

Fig. S18. *Average maximal ToF per nm edge length for all cubes and octahedra across 5 measurement series. Error bars display the standard deviation within each particle type for each series.*

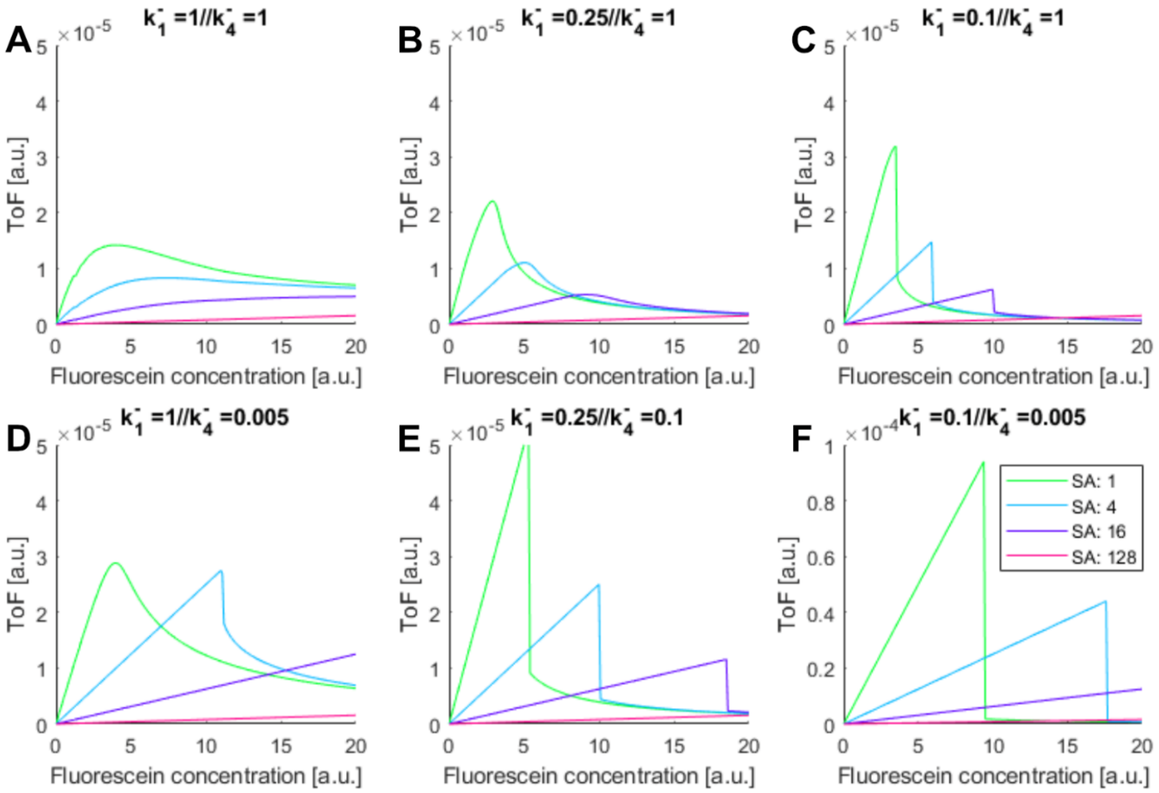

Fig. S19. *Simulated ToFs for varying values of k_1^- or k_4^- for four differently sized particles of relative surface area 1, 4, 16 and 128. (**A**) $k_1^- = 1$ and $k_4^- = 1$. This yields fairly ordinary Langmuir-Hinshelwood profiles. (**B**) $k_1^- = 0.25$ and $k_4^- = 1$. Reactivity decreases for higher fluorescein concentrations due to surface poisoning and increases at low concentrations due to higher surface coverage of fluorescein. (**C**) $k_1^- = 0.1$ and $k_4^- = 1$. Same behavior as in **B** but more drastic. (**D**) $k_1^- = 1$ and $k_4^- = 0.005$. Reactivity increases at low fluorescein concentrations due to more available space for the fluorescein to bind, thus, lowering the probability of the back reaction. (**E**) $k_1^- = 0.25$ and $k_4^- = 0.1$. Both effects combined yield even higher reactivity at low fluorescein concentrations. (**F**) $k_1^- = 0.1$ and $k_4^- = 0.005$. The behavior observed above is even more pronounced, and we see either a very distinct mass transport limited or surface poisoned regime. Note the factor 2 in the y-axis compared to the other panels.*

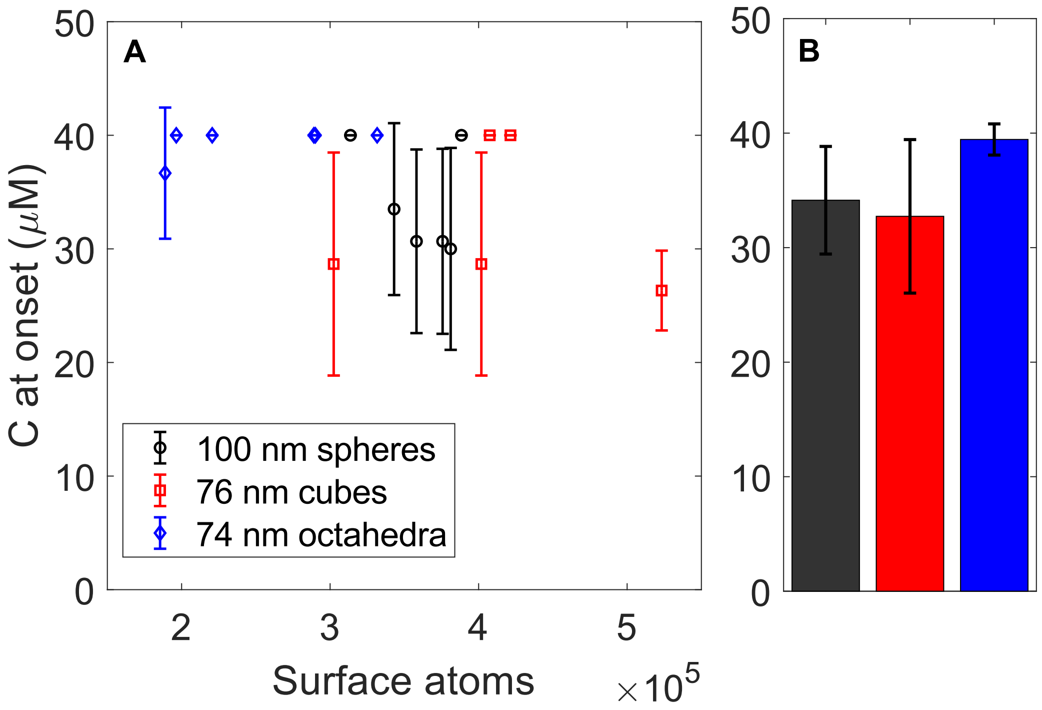

Fig. S20. *(**A**) Concentration (C) at onset of the reaction depicted in **Fig. 4B** for Au faceted spheres, cubes and octahedra, plotted as function of number of estimated surface atoms of each particle. Error bars are standard deviations between three measurements. (**B**) Average values and standard deviation (error bars) between the six faceted spheres, the five cubes and the six octahedra plotted in (A).*

Table S1. Zeta-potentials from three measurements for cubes and octahedra stabilized by CTAC, resulting in a positively charged surface, and PVP/Citrate, resulting in a negatively charged surface, clearly showing the success of the ligand exchange from CTAC to PVP.

Sample	Measurement 1 (mV)	Measurement 2 (mV)	Measurement 3 (mV)	Average (mV)	Standard Deviation (mV)
CTAC Nano-cubes	+43.2	+44.2	+46.7	+44.7	±1.8
PVP/citrate Nano-cubes	-28.9	-28.4	-28.8	-28.7	±0.3
CTAC Nano-octahedra	+50.1	+49.3	+50.7	+50.3	±0.7
PVP/citrate Nano-octahedra	-26.4	-26.2	-25.5	-26.0	±0.5

Video S1.

Video obtained with DFSM when trapping 100 nm Au spheres in the nanofluidic channels with vertical constrictions.
